# Single-cell RNA sequencing of a new transgenic t(8;21) preleukemia mouse model reveals regulatory networks promoting leukemic transformation

**DOI:** 10.1038/s41375-023-02063-z

**Published:** 2023-10-14

**Authors:** Ming Yan, Mengdan Liu, Amanda G. Davis, Samuel A. Stoner, Dong-Er Zhang

**Affiliations:** 1 Moores Cancer Center, University of California San Diego, La Jolla, CA USA; 2 Department of Pathology, University of California San Diego, La Jolla, CA USA; 3 School of Biological Sciences, University of California San Diego, La Jolla, CA USA

**Keywords:** Cancer, Acute myeloid leukaemia

## Abstract

T(8;21)(q22;q22), which generates the AML1-ETO fusion oncoprotein, is a common chromosomal abnormality in acute myeloid leukemia (AML) patients. Despite having favorable prognosis, 40% of patients will relapse, highlighting the need for innovative models and application of the newest technologies to study t(8;21) leukemogenesis. Currently, available AML1-ETO mouse models have limited utility for studying the pre-leukemic stage because AML1-ETO produces mild hematopoietic phenotypes and no leukemic transformation. Conversely, overexpression of a truncated variant, AML1-ETO9a (AE9a), promotes fully penetrant leukemia and is too potent for studying pre-leukemic changes. To overcome these limitations, we devised a germline-transmitted Rosa26 locus AE9a knock-in mouse model that moderately overexpressed AE9a and developed leukemia with long latency and low penetrance. We observed pre-leukemic alterations in AE9a mice, including skewing of progenitors towards granulocyte/monocyte lineages and replating of stem and progenitor cells. Next, we performed single-cell RNA sequencing to identify specific cell populations that contribute to these pre-leukemic phenotypes. We discovered a subset of common myeloid progenitors that have heightened granulocyte/monocyte bias in AE9a mice. We also observed dysregulation of key hematopoietic transcription factor target gene networks, blocking cellular differentiation. Finally, we identified Sox4 activation as a potential contributor to stem cell self-renewal during the pre-leukemic stage.

## Introduction

T(8;21)(q22;q22) is one of the most frequent chromosomal aberrations observed in acute myeloid leukemia (AML) patients [1, 2]. Since most patients respond to induction chemotherapy, t(8;21) patients are considered to have favorable prognosis [1–3]. However, ~40% of these patients will relapse and the median overall survival is only 5 years [1, 4]. Consequently, further research and better t(8;21) models are needed to examine the molecular mechanism driving t(8;21) AML so that more effective therapies can be designed.

T(8;21) joins the N-terminal portion of the AML1 (aka CBFα2, PEBP2αB, or RUNX1) gene on chromosome 21, with almost the entire ETO (aka MTG8 or RUNX1T1) gene on chromosome 8 [5, 6]. AML1 belongs to the RUNX family of transcription factors and plays a critical role during fetal and adult hematopoiesis [7–9]. This protein contains an N-terminal runt homology domain (RHD) for DNA binding and a C-terminal transcriptional regulatory domain (TRD) that recruits additional co-factors necessary for regulation of target gene transcription [10]. ETO cannot directly bind DNA but modulates transcription via interactions with various transcriptional regulators through its four nervy homology regions (NHRs) [11–15]. ETO is not normally expressed in blood cells, such that the t(8;21) translocation only produces the AML1-ETO (AE) fusion protein and not an ETO-AML1 protein [16, 17]. Overall, the AE oncogenic fusion protein, which contains the RHD of RUNX1 and all four NHRs of ETO, drives leukemia development by dysregulating the expression of hematopoietic genes [18–21].

Despite its essential role in t(8;21) pathogenesis, various mouse models reveal that AE expression alone produces mild hematopoietic phenotypes and cannot induce leukemia without cooperating mutations [22]. By contrast, a C-terminally truncated splice variant of AE, AML1-ETO9a (AE9a), can induce leukemia without cooperating mutations in a bone marrow retroviral transduction and transplantation mouse model [23]. AE9a is a naturally occurring isoform [24] detected in most t(8;21) AML patient samples [23, 25–30], making AE9a an attractive alternative model for studying AE leukemogenesis. Unfortunately, AE9a expression is directed by a retroviral long terminal repeat in this model, which dramatically overexpresses the fusion protein in leukemia cells [23, 31]. Additionally, integration of the retroviral cDNA into genomic DNA may activate proto-oncogenes and suppress tumor suppressors, leading to the selective expansion of these clones and leukemogenesis [32, 33]. Consequently, this model also is not conducive for studying pre-leukemic alterations induced by AE9a. Lacking a suitable model, the newest technologies, such as single-cell RNA sequencing (scRNA-seq), have not yet been applied to an in vivo t(8;21) model.

Here, we devised a germline transmitted transgenic Rosa26-AE9a knock-in mouse model that moderately overexpresses the AE9a protein compared to endogenous RUNX1. These mice developed myeloid leukemia without adding cooperative mutations and with longer latency and lower penetrance than mice from the retroviral transduction-transplantation model. Pre-leukemic mice exhibited myeloid progenitor skewing in their bone marrow, with more granulocyte-monocyte progenitors (GMP) at the expense of megakaryocyte-erythroid progenitors (MEP). Additionally, hematopoietic stem and progenitor cells (HSPCs) of pre-leukemic mice had increased clonogenic potential. We performed scRNA-seq on pre-leukemic HSPCs to determine the impact of AE9a expression on HSPC subpopulations and uncover the mechanistic basis of these pre-leukemic phenotypes. First, we confirmed the granulocytic bias of AE9a HSPCs and discovered a small population of common myeloid progenitors (CMPs) directly upstream of MEPs on their differentiation trajectory, which express high levels of GMP signature genes that may alter their cell fate. We also detected alterations to the target gene networks of several key hematopoietic transcription factors, including Cebpa, Cebpe, Gata1, and Tal1, evidence of hindered differentiation in AE9a HSPCs. Finally, we identified Sox4 activation as an important contributor to the enhanced self-renewal of AE9a HSPCs.

## Methods

Please see Supplementary Methods for the complete description of all methods.

### Rosa26-AE9a knock-in transgenic mouse line generation

The CAG-LSL-AE9a-IRES-EGFP-Rosa26 targeting vector was generated by inserting HA-tagged AE9a downstream of the loxP-NEO/STOP-loxP cassette and upstream of the frt-IRES-GFP-frt cassette in the pCAG-STOP-eGFP-RosaTV plasmid described in Piovan et al. [34]. This construct was introduced into C57BL/6J embryonic stem (ES) cells by electroporation. ES cells were screened by PCR of genomic DNA using primers P4/P5. Gene-targeted ES cells were injected into C57BL/6J albino mouse blastocysts and implanted into pseudo-pregnant females to make chimeric founder mice. These founders were backcrossed into the C57BL/6 background using standard techniques to generate the germline transmitted line.

### Animals

All animal protocols were approved by the UCSD Institutional Animal Care and Use Committee (IACUC). C57BL/6J (stock #000664) and Vav-iCre (stock #008610) mice were obtained from Jackson Lab. Leukemic mice were deemed moribund when mice showed signs of illness: reduced activity, hunched back, weight loss, anemia, cachexia, and labored breathing.

## Results

### Generation of a Rosa26 AE9a knock-in transgenic mouse model

**Fig. 1:**
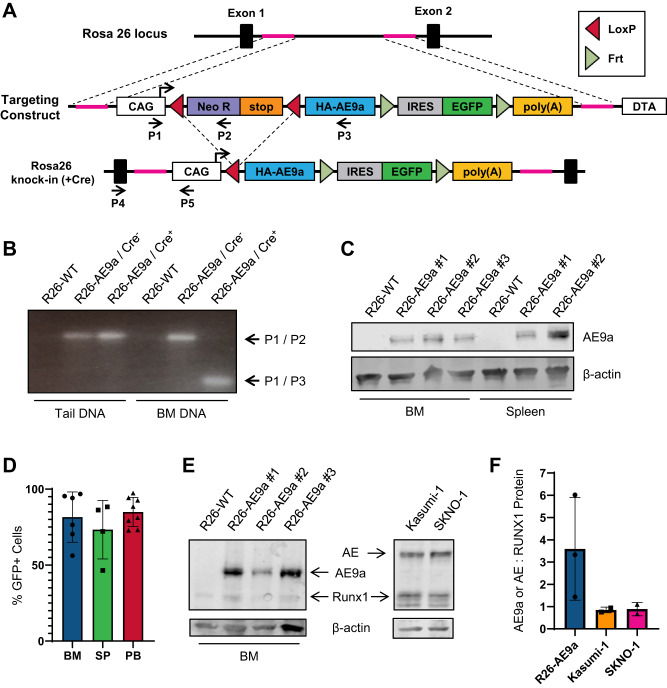
A Rosa26 AE9a KI mouse model moderately overexpresses AE9a protein. **A** Schematic of the Rosa26 locus, AE9a targeting construct, and Rosa26-AE9a knock-in with Vav-iCre-mediated excision. **B** PCR analysis of genomic DNA extracted from the tail or bone marrow (BM) of the indicated mice. A mixture of primers P1, P2, and P3 was utilized. Their binding positions are shown in panel A. P1/P2 produces a band 270 bp in length. P1/P3 produces a band 90 bp in length. **C** Western blot analysis of AE9a (anti-RUNX1 antibody) and β-actin protein (loading control) in bone marrow (BM) or spleen tissue of the indicated mice. **D** Flow cytometric analysis of GFP^+^ cells in the bone marrow (BM), spleen (SP), and peripheral blood (PB) of R26-AE9a mice. BM: n = 6; SP: n = 4; PB: n = 8. **E** Western blot analysis of AE, AE9a, RUNX1 (anti-RUNX1 antibody), and β-actin protein (loading control) in the bone marrow of the indicated mice (left) or t(8;21) cell lines (right). **F** Quantification of the relative AE9a or AE protein to RUNX1 protein from the western blots shown in (**E**). R26-AE9a: n = 3; Kasumi-1: n = 2; SKNO-1: n = 2.

To generate an AE9a germline transmitted transgenic mouse model, we first devised an AE9a and GFP co-expression construct that targets the Rosa26 locus in mice and permits conditional oncogene expression via a floxed Neo-STOP cassette upstream of AE9a (Fig. 1A). We introduced this construct into murine ES cells (Supplementary Fig. 1A). Following generation of a heterozygous knock-in line (Rosa26^AE9a/+^), we crossed these mice with Vav-iCre expressing mice (Vav-iCre^+/−^) for hematopoietic specific recombination. Among progeny, we compared mice with genotypes Vav-iCre^+/−^ Rosa26^AE9a/+^ (R26-AE9a) and Vav-iCre^+/−^ Rosa26^+/+^ (R26-WT). We verified excision of the floxed sequence in bone marrow DNA samples of R26-AE9a mice (Fig. 1B) and observed AE9a protein expression solely in hematopoietic tissues of R26-AE9a mice (Fig. 1C, Supplementary Fig. 1B). Finally, we confirmed that most cells in the bone marrow, spleen, and blood of R26-AE9a mice were expressing the AE9a-GFP transgene (Fig. 1D, Supplementary Fig. 1C).

Considering the importance of oncogene dosage in t(8;21) leukemogenesis [29, 31], we next compared the relative amount of AE9a protein to endogenous RUNX1 protein in R26-AE9a mice. Compared to Kasumi-1 and SKNO-1 human cell lines where the ratio of AE and RUNX1 is close to 1, our mouse models exhibited modest overexpression of AE9a with 3.75x more AE9a than RUNX1 (Fig. 1E, F). Taken together, we successfully generated a germline transmitted R26-AE9a mouse model that expresses moderate AE9a protein levels in the hematopoietic compartment.

### R26-AE9a mice develop AML with long latency and low penetrance

**Fig. 2:**
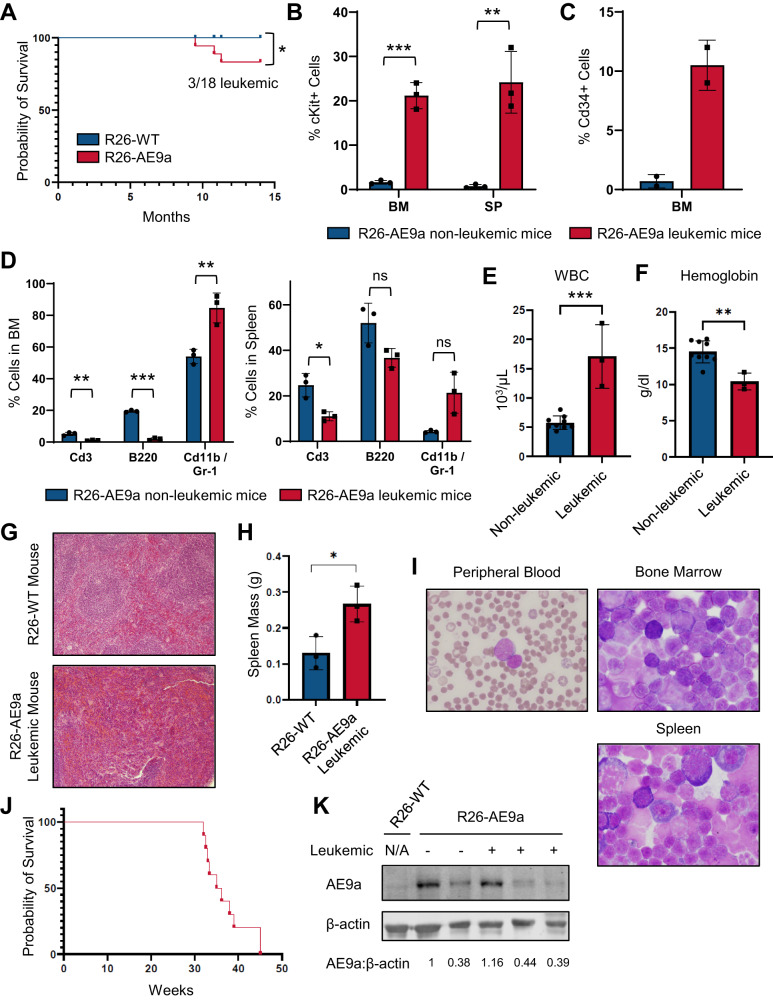
Rosa26-AE9a mice develop AML with long latency and low penetrance. **A** Kaplan-Meier survival curve depicting the proportion of R26-AE9a mice that developed leukemia over the course of 14 months. n = 18 for both R26-WT and R26-AE9a. * *p* < 0.05; one-sided Log-rank (Mantel-Cox) test. **B** Flow cytometric analysis of the percentage of cKit^+^ cells in the bone marrow (BM) and spleen (SP) of non-leukemic and leukemic R26-AE9a mice. Data were collected when leukemic mice were deemed moribund and at 12 weeks of age for non-leukemic mice. Data are mean ± s.d. Non-leukemic mice: n = 3; leukemic mice: n = 3. ** *p* < 0.01, *** *p* < 0.001; student’s *t* tests. **C** Flow cytometric analysis of the percentage of Cd34^+^ cells in the BM of non-leukemic and leukemic R26-AE9a mice. Data were collected at the same time points described in (**B**). Data are mean ± s.d. Non-leukemic mice: n = 2; leukemic mice: n = 2. **D** Flow cytometric analysis of the percentage of Cd3^+^, B220^+^, and Cd11b/Gr-1^+^ cells in the BM (left) and spleen (right) of non-leukemic and leukemic R26-AE9a mice. Data were collected at the same timepoints described in (**B**). Data are mean ± s.d. Non-leukemic mice: n = 3; leukemic mice: n = 3. * *p* < 0.05, ** *p* < 0.01, *** *p* < 0.001; multiple student’s *t* tests with the Holm-Sidak method to correct for multiple comparisons. **E** White blood cell counts in the peripheral blood of the indicated mice. Data were collected when leukemic mice were deemed moribund and at 10 months of age for non-leukemic mice. Data are mean ± s.d. Non-leukemic mice: n = 9; leukemic mice: n = 3. *** *p* < 0.001; student’s *t* test. **F** Hemoglobin levels in the peripheral blood of the indicated mice. Data were collected at the same timepoints described in (**E**). Data are mean ± s.d. Non-leukemic mice: n = 9; leukemic mice: n = 3. ** *p* < 0.01; student’s *t* test. **G** Hematoxylin and eosin (H&E) staining of spleen sections taken from a representative R26-WT mouse and R26-AE9a leukemic mouse. Tissue was collected from the leukemic mice when they were deemed moribund. Age-matched R26-WT mice serve as the control. **H** Spleen weights from the indicated mice. Data were collected in the same manner described in (**G**). n = 3 for both R26-WT and R26-AE9a leukemic mice. * *p* < 0.05; student’s *t* test. **I** Wright-giemsa staining of peripheral blood, bone marrow, and spleen cytospins from a representative R26-AE9a leukemic mouse. **J** Kaplan-Meier survival curve depicting the proportion of mice transplanted with BM from an R26-AE9a leukemic mouse that developed leukemia over the course of the experiment. **K** Western blot analysis of AE9a (anti-RUNX1 antibody) and β-actin protein (loading control) in BM of the indicated mice. Quantifications of AE9a protein relative to β-actin are shown below each lane.

Next, we monitored R26-AE9a mice for signs of disease. R26-AE9a mice developed AML with long latency and low penetrance (Fig. 2A). Mice that developed AML had an expansion of immature cKit^+^ cells in their bone marrow and spleen compared to non-leukemic R26-AE9a mice (Fig. 2B, Supplementary Fig. 2A). These mice also had more Cd34^+^ cells in their bone marrow (Fig. 2C, Supplementary Fig. 2B) in agreement with t(8;21) patient phenotypes [35]. Some leukemic cells in the bone marrow and spleen expressed mature lineage markers, with more granulocytic marker expression and less lymphocytic marker expression (Fig. 2D, Supplementary Fig. 2C). In the peripheral blood of leukemic mice, we observed significant white blood cell expansion (Fig. 2E) and hemoglobin reduction (Fig. 2F), trends commonly seen in t(8;21) patients [36]. Normal spleen and liver morphology were also disturbed by the invasion of immature myeloid cells (Fig. 2G, Supplementary Fig. 2D), and R26-AE9a leukemic spleens were significantly larger than R26-WT spleens (Fig. 2H). Blast-like cells were observed in the peripheral blood, bone marrow, and spleen (Fig. 2I). We quantified this myeloid blast expansion in the bone marrow, supporting the AML diagnosis (Supplementary Table 1). Indeed, these leukemic cells were transplantable and produced fully penetrant leukemia in recipient mice (Fig. 2J). Finally, we measured AE9a protein levels. Leukemic mice expressed similar levels of AE9a protein in their bone marrow compared to mice that did not develop leukemia (Fig. 2K), demonstrating that AE9a overexpression is not required for leukemia development in the current model (Supplementary Fig. 2E).

We next tested whether perturbation of R26-AE9a hematopoietic stem cells (HSCs) via transplantation could accelerate or heighten disease development. Therefore, we isolated total bone marrow from R26-AE9a mice at 12 weeks old and transplanted the cells into lethally irradiated recipient mice (Supplementary Fig. 3A). Transplanted mice also developed AML after a long latency period with modestly higher penetrance than the R26-AE9a model (Supplementary Fig. 3B). Transplanted mice that developed AML had more cKit^+^ cells (Supplementary Fig. 3C) and blast-like cells present in the bone marrow and spleen (Supplementary Fig. 3D). Taken together, this germline transmitted R26-AE9a mouse model more closely represents human t(8;21) AML development than our previously established AE9a retroviral transduction-transplantation model with less fusion protein expression, lower penetrance, and longer latency.

### R26-AE9a pre-leukemic mice have altered HSPC frequencies

**Fig. 3:**
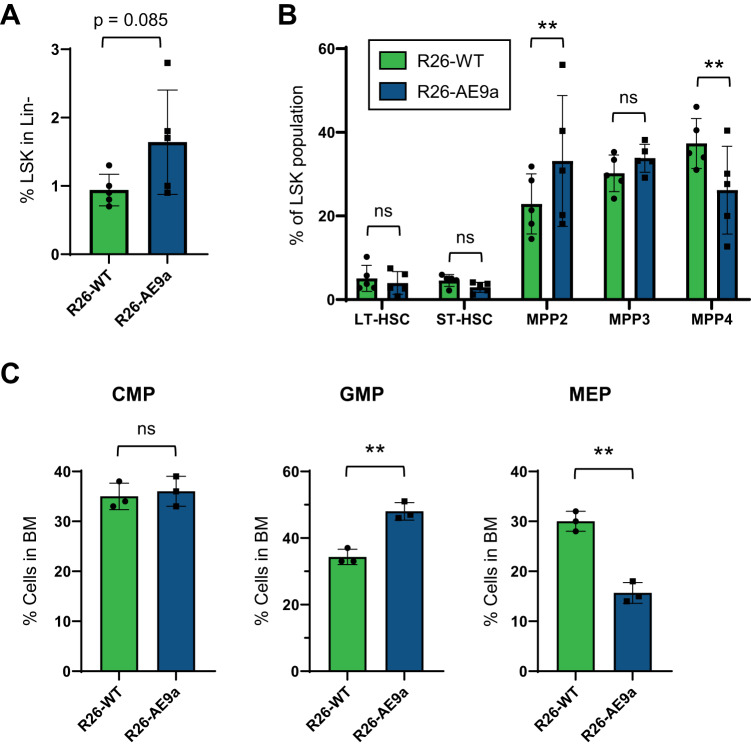
The stem and progenitor cell compartment is altered in Rosa26-AE9a pre-leukemic mice. **A** Flow cytometric analysis of the percentage of Lin^-^Sca-1^+^cKit^+^ (LSK) cells in the Lin^-^ bone marrow (BM) of R26-WT and R26-AE9a pre-leukemic mice at 12 weeks. Data are mean ± s.d. R26-WT mice: n = 5; R26-AE9a mice: n = 5. *p* = 0.085; student’s *t* test. **B** Flow cytometric analysis of the percentage of long-term (LT) and short-term (ST) hematopoietic stem cell (HSC) populations and various multipotent progenitor (MPP) populations among Lin^-^Sca-1^+^cKit^+^ (LSK) cells in the BM of R26-WT and R26-AE9a pre-leukemic mice at 12 weeks. LT-HSC: Flk2^−^/CD150^+^/CD48^−^LSK; ST-HSC: Flk2^−^/CD150^−^/CD48^−^LSK; MPP2: Flk2^−^/CD150^+^/CD48^+^LSK; MPP3: Flk2^−^/CD150^−^/CD48^+^LSK; MPP4: Flk2^+^/CD150^−^/CD48^+^LSK. Data are mean ± s.d. R26-WT mice: n = 5; R26-AE9a mice: n = 5. ** *p* < 0.01; two-way ANOVA with post-hoc Fisher’s Least Significant Difference (LSD) test. **C** Flow cytometric analysis of the percentage of common myeloid progenitors (CMP), granulocyte-monocyte progenitors (GMP), and megakaryocyte-erythroid progenitors (MEP) in the BM of R26-WT and R26-AE9a pre-leukemic mice at 12 weeks. Data are mean ± s.d. R26-WT mice: n = 3; R26-AE9a mice: n = 3. ** *p* < 0.01; student’s *t* tests.

To characterize the impact of AE9a on leukemia development, we next examined the frequencies of individual hematopoietic cell populations in the bone marrow of 12-week-old R26-WT and pre-leukemic R26-AE9a mice. We observed an increased (Lin^-^Sca1^+^cKit^+^) LSK frequency in the R26-AE9a mice compared to R26-WT mice, though the data are statistically insignificant (Fig. 3A). Within this LSK fraction, we measured the frequencies of primitive HSC and multipotent progenitor (MPP) populations and detected a significant increase in the percentage of MPP2 cells with a concurrent, significant decrease in MPP4 cells in R26-AE9a mice compared to R26-WT mice (Fig. 3B, Supplementary Fig. 4A). MPP2 cells are myeloid-biased progenitors with granulocyte/monocyte (GM) and megakaryocytic lineage potential, whereas MPP4 cells are lymphoid-biased progenitors [37, 38]. These results indicate that R26-AE9a mice are beginning to exhibit HSPC alterations that contribute to myeloid expansion. Next, we examined committed progenitor populations and saw a significant increase in granulocyte-monocyte progenitor (GMP) frequency at the expense of megakaryocyte-erythroid progenitor (MEP) frequency, confirming dysregulation of progenitor cells in pre-leukemic R26-AE9a mice (Fig. 3C, Supplementary Fig. 4B).

We also analyzed mature blood cell frequencies in R26-WT and R26-AE9a pre-leukemic mice. We monitored peripheral blood composition over 11 months and observed no difference in total white blood cells, red blood cells, or platelets (Supplementary Fig. 5A). There was a moderate trend towards granulocytes at the expense of lymphocytes, however the difference was not statistically significant. Likewise, there were similar frequencies of T-cells, B-cells, and granulocytes in the bone marrow and spleens of 12-week-old mice. (Supplementary Fig. 5B, C).

### R26-AE9a progenitor cells have enhanced clonogenic potential in vitro

**Fig. 4:**
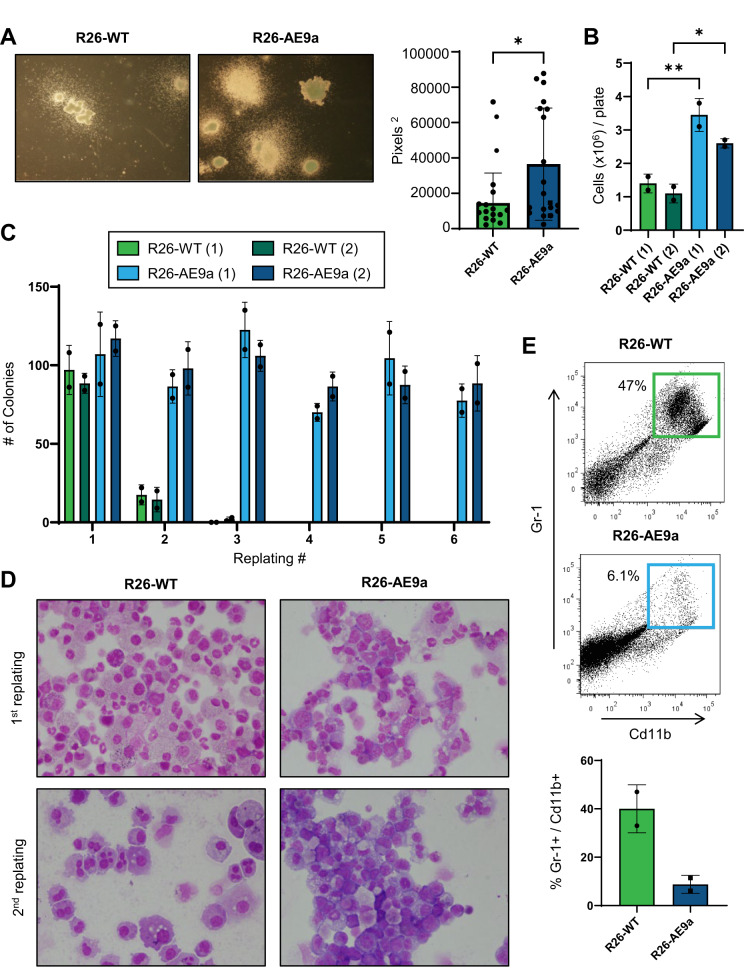
Rosa26-AE9a progenitor cells have increased clonogenic potential in vitro. **A** (Left) Representative images of colonies formed in methylcellulose by R26-WT and R26-AE9a Lin^−^cKit^+^ (LK) cells. (Right) Quantification of the main colony body sizes in representative images of all experimental replicates. R26-WT: n = 15; R26-AE9a: n = 19. * *p* < 0.05; student’s *t* test. **B** The total number of cells per plate following colony formation of R26-WT and R26-AE9a LK cells. Two biological replicates for each condition are displayed as separate bars. Data are mean ± s.d. of technical duplicates. * *p* < 0.05, ** *p* < 0.01. Unpaired student’s *t* tests. **C** Number of colonies formed upon sequential replating of 40,000 cells/plate of R26-WT and R26-AE9a cells. 5000 LK cells per plate were seeded for colony formation prior to the initial replating. Two biological replicates for each condition are displayed as separate bars. Data are mean ± s.d. of technical duplicates. **D** Wright-Giemsa staining of R26-WT and R26-AE9a cytospins collected from the colonies present after the 1st and 2nd replating. **E** Flow cytometric analysis of Cd11b and Gr-1 cell surface markers on R26-WT and R26-AE9a cells derived from colonies formed after the 1st replating. The plots on top are quantified in the bar graph on the bottom. Data are mean ± s.d. R26-WT: n = 2; R26-AE9a: n = 2.

Considering these altered HSPC subpopulation frequencies, we hypothesized that the R26-AE9a pre-leukemic HSPCs may be functionally divergent from their control counterpart. To test this hypothesis, we measured the colony-forming ability of Lin^-^cKit^+^ (LK) cells isolated from 12-week-old R26-WT and R26-AE9a mice. R26-AE9a LK cells produced bigger colonies than R26-WT LK cells (Fig. 4A), resulting in significantly higher numbers of total cells per plate (Fig. 4B). We next performed a serial replating assay to measure the clonogenic potential of R26-AE9a and R26-WT LK cells. R26-AE9a LK cells replated over the course of the 6-week experiment, producing similar numbers of colonies after each replating (Fig. 4C). By contrast, R26-WT cells replated only once and formed very few colonies compared to the initial plating. Additionally, R26-AE9a LK colonies contained cells with a distinct blast-like morphology in stark contrast to the differentiated granulocytic cell morphology of R26-WT LK colonies (Fig. 4D). Indeed, there was a significantly higher percentage of Gr-1/Cd11b^+^ cells in R26-WT colonies compared to R26-AE9a colonies (Fig. 4E). Altogether, these findings demonstrate that R26-AE9a HSPCs are already exhibiting signs of blocked differentiation and increased clonogenic potential at only 12 weeks.

### scRNA-seq shows enhanced GMP lineage priming in LK cells of R26-AE9a pre-leukemic mice

Since phenotypes from this new t(8;21) mouse model resembled aspects of human t(8;21) AML, we wondered whether gene expression changes were also similar. Therefore, we performed scRNA-seq of LK cells isolated from 12-week-old R26-AE9a and R26-WT mice and identified differentially expressed genes by pseudobulk analysis [39]. We compared these genes to differentially expressed genes in human t(8;21) Kasumi-1 cells with and without AE and identified 385 overlapping differentially expressed genes (Supplementary Fig. 6A). Among gene-disease association datasets [40, 41], AML-M2 genes were most significantly enriched in this overlapping set of genes; t(8;21) AML belongs to this AML subcategory (Supplementary Fig. 6B). Additionally, gene ontology (GO) analysis [41] revealed enrichment of several cancer-related and immune activation pathways (Supplementary Fig. 6C). These observations highlight similarities between this new t(8;21) mouse model and human t(8;21) AML, suggesting that studying gene expression alterations in this new mouse model may provide valuable insights into human disease.

**Fig. 5:**
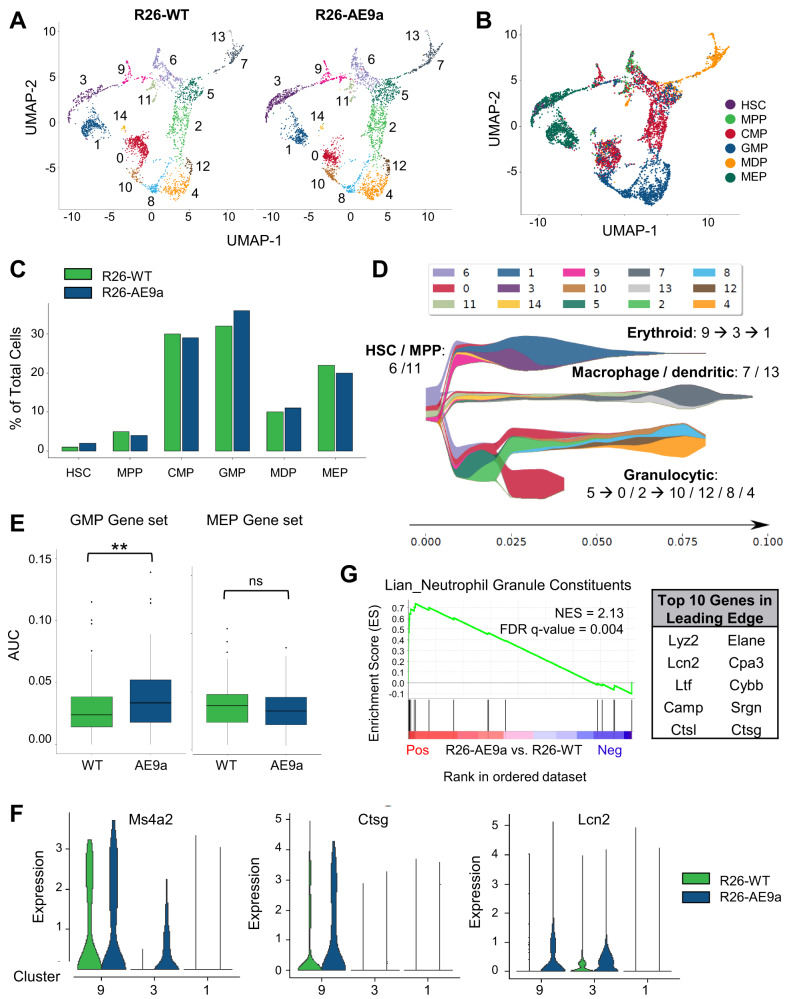
Single-cell RNA sequencing reveals enhanced GMP lineage priming in LK cells of AE9a pre-leukemic mice. **A** Uniform Manifold Approximation and Projection (UMAP) of Lin^−^cKit^+^ (LK) cells isolated from the bone marrow (BM) of R26-WT (left) and R26-AE9a (right) mice at 12 weeks. Fifteen cell clusters were identified and differentiated by color. **B** UMAP of LK cells isolated from the BM of R26-WT and R26-AE9a mice (combined) colored by cell types assigned via cellHarmony analysis. HSC hematopoietic stem cells, MPP multipotent progenitors, CMP common myeloid progenitors, GMP granulocyte-monocyte progenitors, MDP monocyte-dendritic cell progenitors, MEP megakaryocyte-erythroid progenitors. **C** Bar graph depicting the percentages of each assigned cell type among all sequenced LK cells from BM of either R26-WT or R26-AE9a mice. **D** STREAM plot depicting the differentiation trajectory of all sequenced R26-WT LK cells. Cluster numbers and colors reflect those assigned in (**A**). The cell types differentiating in each branch are labeled and the cluster order along the differentiation trajectory of each branch is listed (immature → mature). At a given pseudotime, width of a branch is proportional to the total number of cells and width of colors within a branch is proportional to the relative abundance of the corresponding cells. **E** AUCell analysis of the enrichment of both GMP and MEP gene sets in R26-WT and R26-AE9a cluster 9 LK cells. ** *p* < 0.01; student’s *t* test. **F** Violin plots showing the distribution of gene expression across all cells within the indicated samples (R26-WT or R26-AE9a) and clusters (9, 3, or 1). **G** Plot from gene set enrichment analysis (GSEA) of differentially expressed genes between R26-WT and R26-AE9a LK cells within cluster 9. The Lian Neutrophil Granule Constituents dataset is shown. The top 10 upregulated genes in R26-AE9a cells that comprise the leading edge of the enrichment are shown in the table to the right.

To further characterize the composition and functional changes of R26-AE9a pre-leukemic HSPCs, we performed Uniform Manifold Approximation and Projection (UMAP) of the scRNA-seq dataset, revealing 15 distinct clusters in these mice (Fig. 5A). To uncover the cell types within each cluster, we used cellHarmony to unbiasedly label each cell based on its transcript expression profile (Fig. 5B) [42]. This analysis revealed the divergence of MEP, GMP, and monocyte-dendritic cell progenitor (MDP) lineages in the three main arms of the UMAP. We confirmed the validity of these cell labels by quantifying expression of established marker genes (Supplementary Fig. 7A–D). Next, we compared the frequencies of these cell populations and observed a modest increase of GMPs with a concurrent decrease of MEPs in R26-AE9a mice (Fig. 5C), in agreement with our flow cytometric analysis (Fig. 3C).

Considering these consistent data regarding GMP and MEP skewing, we hypothesized that immature cells may be biased towards granulocytes and monocytes at the expense of erythrocytes and megakaryocytes in R26-AE9a pre-leukemic mice. To address this hypothesis, we first mapped the differentiation trajectory of cells within our R26-WT mice. Pseudotime analysis revealed three distinct branches of differentiation representing the separation into more restricted MEPs, GMPs, and MDPs (Fig. 5D) [43]. For MEP development, cluster 9 was most immature, followed by cluster 3, and finally cluster 1 (Supplementary Fig. 8A). Importantly, cluster 9 consists primarily of CMPs, whereas clusters 3 and 1 contain predominantly MEPs (Supplementary Fig. 8B). Therefore, we reasoned that cluster 9 could be responsible for the lineage skewing in our R26-AE9a pre-leukemic mice. To identify differences in lineage priming of cells within this population, we examined the enrichment of MEP and GMP gene signatures [44] using the Area Under the Curve (AUC) method. Indeed, R26-AE9a cluster 9 cells expressed significantly higher GMP gene set scores than R26-WT cells and a trend towards decreased MEP gene set scores (Fig. 5E). Upregulated GMP signature genes included Ms4a2, Ctsg, and Lcn2 (Fig. 5F). In agreement with this observation, GSEA of differentially expressed genes in cluster 9 revealed an enrichment of genes associated with neutrophil granules in R26-AE9a cells (Fig. 5G).

Next, we examined the GMP branch of our pseudotime analysis. For GMP development, cluster 5 is most immature followed by clusters 2 and 0, and finally clusters 8, 10, 12, and 4. (Fig. 5D**;** Supplementary Fig. 8C, D). We examined GMP and MEP signatures in cluster 5 by AUC and saw no significant difference (Supplementary Fig. 9A). Similarly, there is no significant difference in cluster 6, which is upstream of both branches and has the highest proportion of CMPs among all clusters (Supplementary Fig. 9B). We conclude that in R26-AE9a pre-leukemic mice, cells on the trajectory to becoming MEPs have not solely committed to their MEP fate but are also primed towards a potential GMP fate, resulting in the observed lineage skewing.

### scRNA-seq reveals blocked differentiation in LK cells of R26-AE9a pre-leukemic mice

**Fig. 6:**
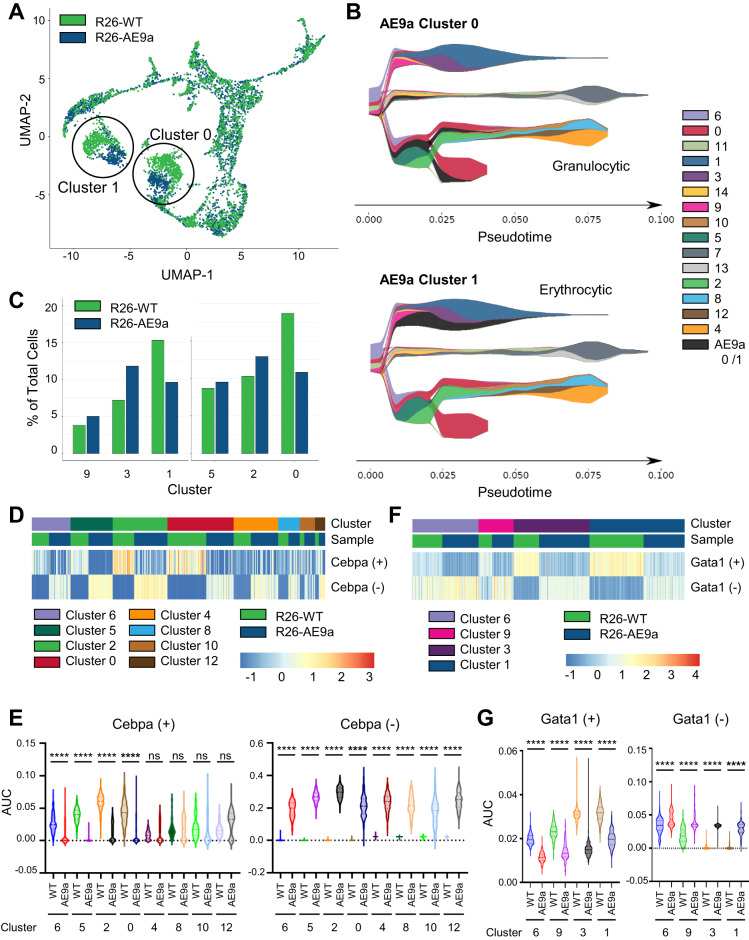
Single-cell RNA sequencing reveals cellular immaturity of LK cells in AE9a pre-leukemic mice. **A** Uniform Manifold Approximation and Projection (UMAP) of Lin^−^cKit^+^ (LK) cells isolated from the bone marrow (BM) of R26-WT (green) and R26-AE9a (blue) mice at 12 weeks. Cell positions differ between R26-WT and R26-AE9a LK cells most dramatically in the circled clusters 0 and 1. **B** STREAM plots depicting the differentiation trajectory of all sequenced R26-WT LK cells. R26-AE9a cluster 0 cells (top) and cluster 1 cells (bottom) are mapped onto the R26-WT trajectory and are both shown in black. At a given pseudotime, width of a branch is proportional to the total number of cells and width of colors within a branch is proportional to the relative abundance of the corresponding cells. **C** Bar graph depicting the percentages of cells in each cluster among all sequenced LK cells from BM of either R26-WT or R26-AE9a mice. **D** Heatmap depicting regulatory network (regulon) analysis of LK cells in the indicated clusters and samples. Row Z-scored AUCell enrichment scores for Cebpa positive and negative regulons within each cell are shown. **E** Violin plots quantifying the data depicted in (**D**). **** *p* < 0.0001; Kruskal-Wallis test with post-hoc uncorrected Dunn’s test. **F** Heatmap depicting regulatory network (regulon) analysis of LK cells in the indicated clusters and samples. Row Z-scored AUCell enrichment scores for Gata1 positive and negative regulons within each cell are shown. **G** Violin plots quantifying the data depicted in (**F**). **** *p* < 0.0001; Kruskal-Wallis test with post-hoc uncorrected Dunn’s test.

When comparing the UMAP projections for R26-AE9a pre-leukemic mice and R26-WT mice, two clusters showed a clear separation of cells across the two sample conditions: clusters 0 and 1 (Fig. 6A). To understand the basis of the difference between these two clusters, we mapped both aberrant R26-AE9a clusters onto the R26-WT pseudotime to examine their relative cell maturity. Interestingly, both clusters have distributions that appear earlier than their control counterparts, suggesting that R26-AE9a clusters 0 and 1 contain cells that are more immature (Fig. 6B). Consistent with this notion, there are also fewer cells in clusters 0 and 1 in the R26-AE9a pre-leukemic LK population compared to R26-WT and more cells in immature, upstream clusters 9, 3, 5, and 2 (Fig. 6C**)**. We further examined the differences in gene expression between R26-WT and R26-AE9a cells of both clusters 0 and 1 by performing a sub clustering analysis. Cluster 0 cells could be subdivided into four distinct subclusters, with R26-AE9a cells residing almost exclusively in subcluster 0 (Supplementary Fig. 10A, B). R26-WT subclusters 2-3 express granulocytic gene signatures, whereas subcluster 1 expresses monocytic genes (Supplementary Fig. 10C). GSEA comparing the R26-AE9a subcluster 0 with the granulocytic R26-WT subclusters 2–3 showed a decrease in mature granulocytic genes and enrichment of AE targets, supportive of a differentiation block (Supplementary Fig. 10D). Comparison to monocytic subcluster 1, however, revealed a shift in cellular identity towards an immature granulocytic gene expression profile (Supplementary Fig. 10E). Similarly, we performed subcluster analysis of cluster 1 in the MEP branch. Cluster 1 could be subdivided into three subclusters, with R26-AE9a cells residing mostly in subcluster 1 (Supplementary Fig. 11A, B). Interestingly, many of the marker genes associated with the R26-AE9a subcluster 1 were granulocytic in nature (Supplementary Fig. 11C), an observation that was confirmed by GSEA analysis comparing subcluster 1 with subclusters 0 and 2 (Supplementary Fig. 11D). Altogether, these findings support the conclusion that R26-AE9a mice exhibit a granulocytic bias and further suggest that these cells are more immature than their R26-WT granulocytic progenitor counterparts.

Next, we performed regulon analysis across clusters in both the GMP and MEP branches [45]. During GMP differentiation, we observed both downregulation of the Cebpa positive regulon and upregulation of the Cebpa negative regulon in stem cell cluster 6 and upstream clusters 5, 2, and 0, indicating reduced activation of Cebpa, a direct AE target gene [46] (Fig. 6D, E). Indeed, we measured significant downregulation of Cebpa expression in cluster 5 and modest, insignificant downregulation in clusters 6 and 2 (Supplementary Fig. 12A). In downstream clusters 4, 8, 10, and 12, Cebpa negative regulon genes were also significantly upregulated, whereas Cebpa positive regulon genes were not significantly altered. Cebpa is not strongly expressed in these downstream clusters, but there was still an insignificant trend of downregulation in clusters 8 and 12. In addition to these Cebpa regulon alterations, we observed downregulation of the Cebpe positive regulon, a critical transcription factor for granulocyte differentiation (Supplementary Fig. 12B–D). The downregulation of this regulon was not as severe as that of Cebpa but nonetheless supports a blocked differentiation phenotype. In the erythroid differentiation branch, we similarly observed downregulation of the positive regulons and upregulation of the negative regulons of Gata1 and Tal1, crucial transcription factors for erythrocyte maturation (Fig. 6F, G**;** Supplementary Fig. 12E, F**)**. Neither is confirmed as a direct AE target gene; consequently, we did not detect any difference in expression of these regulators in R26-AE9a cells versus R26-WT cells (Supplementary Fig. 12G). Taken together, moderate AE9a expression in LK cells begins blocking differentiation well in advance of leukemia development.

### Sox4 activation may contribute to the enhanced self-renewal of R26-AE9a HSPCs

**Fig. 7:**
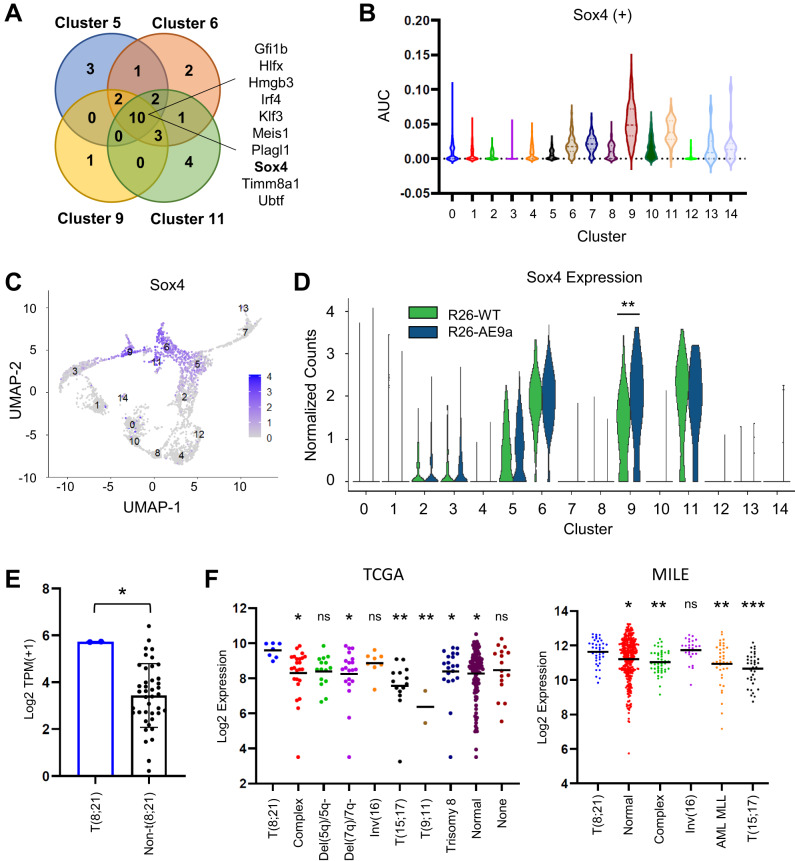
Sox4 is activated in LK cells of AE9a pre-leukemic mice. **A** Venn diagram depicting the positive regulatory networks (regulons) that were significantly up or only detected in R26-AE9a cells versus R26-WT cells of the indicated clusters. The listed positive regulons were common to all four clusters examined. **B** Violin plots depicting AUCell enrichment scores for the Sox4 positive regulon within each cell across all clusters in R26-AE9a mice. **C** Feature plot depicting Sox4 expression across all sequenced R26-AE9a LK cells, organized by Uniform Manifold Approximation and Projection (UMAP). **D** Violin plots showing the distribution of Sox4 gene expression across all cells within the indicated samples (R26-WT or R26-AE9a) and clusters. ** *p* < 0.01; Kruskal-Wallis test with post-hoc uncorrected Dunn’s test. **E** Bar graph depicting normalized Sox4 expression [log2 TPM( + 1)] in all AML cell lines from the DepMap database (depmap.org). Kasumi-1 and SKNO-1 are the two t(8;21) cell lines. * *p* < 0.05; student’s *t* test. **F** Scatter plots depicting normalized Sox4 expression in two datasets from the Bloodspot database [61]. The Cancer Genome Atlas (TCGA): Affymetrix probe 201417_at. Microarray Innovations in Leukemia (MILE): GSE13159; Affymetrix probe 201416_at. Complex, complex aberrant karyotype. Normal, normal karyotype. * *p* < 0.05; ** *p* < 0.01; *** *p* < 0.001. One-way ANOVA with post-hoc Fisher’s Least Significant Difference (LSD) test. Asterisks above each group of patients represent the significance of that group compared to the t(8;21) patient group. In the TCGA dataset, patients in the “Del(5q)/5q-“ (*p* = 0.0564) and “None” (*p* = 0.0663) groups were almost significant.

Finally, we examined upstream clusters 6, 11, 5, and 9 for regulon activation that may contribute to the enhanced self-renewal ability of R26-AE9a HSPCs. We focused on positive regulons that were only detected or significantly higher in R26-AE9a cells of all four of these clusters (Fig. 7A). Among the identified regulons, we were most intrigued by that of Sox4, a transcription factor that enhances HSC self-renewal [47] and induces leukemia when overexpressed [48–50]. The Sox4 regulon was solely detected, not only in these four clusters but in all clusters, though activation tended to be higher in clusters representing more immature cell types (6, 9, and 11) (Fig. 7B) where Sox4 expression was most prominent (Fig. 7C). Additionally, we observed significant upregulation of Sox4 in R26-AE9a cluster 9 cells versus R26-WT cells and modest, insignificant upregulation in ten of the fifteen clusters (Fig. 7D). In support of Sox4 activation in R26-AE9a HSPCs, we observed upregulation of genes in the Sox4 positive regulon in all four upstream clusters (Supplementary Fig. 13A). Finally, we wondered whether Sox4 activation might also be observed in human t(8;21) cells. Indeed, t(8;21) cell lines Kasumi-1 and SKNO-1 had significantly higher Sox4 expression than non-t(8;21) AML cell lines (Fig. 7E) and t(8;21) patients had significantly higher Sox4 expression compared to most non-t(8;21) patient groups in two publicly-available datasets (Fig. 7F). Altogether, we have identified an interesting candidate gene that may contribute to the self-renewal of AE9a-expressing cells.

## Discussion

Here, we devised a Rosa26 AE9a knock-in mouse model that is distinct from previous t(8;21) models and suitable for studying pre-leukemic changes. Modeling and studying t(8;21) leukemogenesis in vivo has been historically challenging because cooperating mutations are required alongside AE for transformation [22]. Discovery of the naturally occurring, truncated AE9a isoform was promising because it produced fully penetrant leukemia in a bone marrow retroviral transduction and transplantation mouse model [23]. However, this model was too potent for studying pre-leukemic changes because onset of leukemia was fast and unphysiological overexpression was required for transformation [31]. Additionally, the random nature of retrovirus integration sites may enhance proto-oncogene function or disrupt tumor suppressor function, promoting leukemia independent of the effects of AE9a [32, 33]. Our model overcomes these limitations: Rosa26-AE9a knock-in mice produced leukemia without cooperating mutations, with long latency, and with a modestly elevated AE9a dosage that was unchanged in leukemic versus non-leukemic mice. Leukemic mice had a higher percentage of Cd34^+^ cells in their bone marrow and leukemic cells expressed mature granulocytic cell surface markers, both features of t(8;21) patients that were not observed in the AE9a transduction-transplantation model [35, 51, 52]. Additionally, leukemic mice had more white blood cells and reduced hemoglobin levels, trends commonly seen in t(8;21) patients that were more severe in the transduction-transplantation model [23, 36]. Furthermore, leukemic mice in the current R26-AE9a model and previous transduction-transplantation model both exhibited splenomegaly and infiltration of myeloblasts into the liver and spleen [23]. R26-AE9a pre-leukemic mice also exhibited several phenotypes that contribute to t(8;21) leukemogenesis: modestly increased LSK cell frequencies, skewing towards GMPs at the expense of MEPs, and increased clonogenic potential. Notably, Agrawal et al. recently reported a Rosa26 AE9a knock-in model that also exhibited increased clonogenic potential of AE9a-expressing LK cells and an expansion of GMPs in the bone marrow of R26-AE9a mice [29]. However, AE9a expression was lower in their model compared to ours and none of their mice developed leukemia. This may indicate a link between oncogene dosage and disease manifestation. But considering the low penetrance and lack of AE9a upregulation in leukemic mice of our model, we suspect that cooperating mutations may also contribute to leukemic transformation.

After establishing this new t(8;21) mouse model, we performed scRNA-seq on R26-WT and R26-AE9a pre-leukemic mice to uncover the mechanistic basis of the observed phenotypes. First, we noticed that the myeloid progenitor skewing we measured via bulk flow cytometric analysis was recapitulated at the single cell level. This AE-induced skewing away from MEPs and toward GMPs has been documented by several other groups [53–56] and matches the M2 classification of t(8;21) leukemia, characterized by an overproduction of granulocytic precursors. In R26-AE9a mice, we identified a cluster of predominantly CMPs (cluster 9) directly upstream of MEPs on their differentiation trajectory that expressed higher levels of GMP signature genes than the corresponding cluster in R26-WT mice. These data indicate that a subset of CMPs which are primed to become MEPs may be reprogrammed by AE9a to express genes that prompt these CMPs to adopt a GMP cell fate. A previous report regarding this progenitor reprogramming event implicated downregulation of Scl (aka Tal1) and Gata1 with concurrent upregulation of Pu.1 as major drivers of this shift [53]. Similarly, we observed downregulation of Tal1 and Gata1 activity, evidenced by alterations to their positive and negative regulons in the MEP branch, though the effect was not more potent in cluster 9 than the others.

Our scRNA-seq data also revealed blocked differentiation, a canonical function of AE in t(8;21) leukemogenesis [57]. In our dataset, clusters 1 and 0 of the MEP and GMP differentiation branches noticeably diverged in R26-AE9a versus R26-WT cells. R26-AE9a cells in these clusters adopted a granulocytic cell identity, further supporting the granulocytic bias in these mice, and were also more immature than their granulocytic R26-WT counterparts. Mechanistically, we identified altered regulatory networks of several critical transcription factors that contribute to this differentiation block. Though none of these factors is new, our scRNA-seq data provided insight regarding which cell populations exhibit AE9a-induced regulon disturbances, highlighting the importance of cell context. For example, Cebpa, a directly downregulated AE target gene, showed more dramatic positive regulon downregulation in upstream clusters containing predominantly CMPs (6, 5, 2, and 0) than in downstream clusters containing mostly GMPs (4, 8, 10, 12). This could be because Cebpa expression is lower in these downstream clusters such that downregulation of Cebpa positive targets is harder to detect. Conversely, negative Cebpa targets seemed to be much more sensitive to modest changes in Cebpa activity and may contribute more heavily to the gene expression profile of AE9a-expressing GMPs. In addition to these nuanced cell context differences, our dataset also allowed us to compare the effects of AE9a expression on direct transcriptional target genes versus non-target genes and their downstream transcriptional networks. Collectively, the degree of regulon dysregulation tended to be more potent for the target gene Cebpa than for the non-target genes Gata1 and Tal1. Interestingly, we did not see a dramatic change in expression of these non-target transcription factors, but instead their downstream target networks, suggesting that in addition to directly altering target gene expression, a major function of AE9a may be disrupting transcription factor function and/or target gene networks via protein-protein interactions or other post-transcriptional mechanisms. In agreement with this hypothesis, a previous report showed that AE blocks the acetylation of Gata1, inhibiting its function during erythroid differentiation [56].

Finally, we identified several regulons that were only detected or significantly more active in R26-AE9a upstream cell clusters 6, 11, 5, and 9. Among these regulons, we were most intrigued by Sox4 because it is implicated in HSC self-renewal [47] and overexpression induces leukemia [48–50]. Consequently, we believe that Sox4 may contribute to the self-renewal capacity of R26-AE9a HSPCs. In support of this hypothesis, a previous report revealed that Sox4 is upregulated in Cebpa deficient cells and contributes to their replating ability [58]. Since Cebpa is a directly downregulated AE target [46], we might also expect to see Sox4 activation in AE expressing cells. Indeed, we observed modest upregulation in most AE9a clusters and previous studies revealed Sox4 upregulation upon AE induction in human cells [59, 60]. Furthermore, we noted that Sox4 is highly expressed in t(8;21) AML cell lines compared to most AML cell lines and t(8;21) patients express high levels of Sox4 compared to non-t(8;21) AML patients. Collectively, these data make a strong case for further studies regarding the role of Sox4 in the self-renewal of AE cells.

In conclusion, we devised a new preleukemia t(8;21) mouse model that overcomes the major limitations of previous models, utilized scRNA-seq to define differences in HSPC subpopulations that contribute to hallmark AE phenotypes, and identified a promising candidate gene mediating the enhanced clonogenic potential.

### Supplementary information

Yan_et al_AE9a_Revised Supplementary Data_2023.pdf

## Data Availability

The scRNA-seq data has been uploaded to the Gene Expression Omnibus (GEO) under accession GSE173712. The DNA construct generated for this study will be made available upon email request to the corresponding author.
