# Supplementary material for: Single-cell RNA sequencing of a new transgenic t(8;21) preleukemia mouse model reveals regulatory networks promoting leukemic transformation
Source: Leukemia. 2023 Oct 14;38(1):31–44. doi: 10.1038/s41375-023-02063-z (PMC10776403; doi:10.1038/s41375-023-02063-z)

SUPPLEMENTARY METHODS

Hematopoietic cell collection and tissue processing

Bone marrow cells were harvested from two femurs and two tibias per mouse. Cells were flushed from these bones using cold PBS and syringes attached to 21-gauge needles. Splenocytes were obtained by physical tissue disruption and pipetting in cold PBS. Red blood cells were lysed from each source by resuspending the cells in cold ACK buffer (0.1 mM Na_2EDTA, 10 mM $KHCO_3$, 150 mM NH_4Cl), incubating for 5 minutes, and washing with cold PBS. Finally, cells were passed through a 40 µM cell strainer and were ready for analysis.

Cells from liver and kidney tissue were obtained by physical tissue disruption and pipetting in cold PBS.

Bone marrow transplantation

AE9a retroviral transduction and bone marrow transplantation (BMT) was performed exactly as previously described[1].

For transplantation of R26-AE9a bone marrow into secondary recipients, total bone marrow from three mice was harvested as described when the mice were 12 weeks old. These cells were resuspended in PBS at a density of 1 x 10^7 cells/mL and 100 µL of cell suspension was injected into the tail veins of lethally irradiated recipient mice (9.5 Gy, 6-8 hours pre-transplantation).

For leukemic cell secondary BMT, 5 x 10^5 bone marrow cells from an R26-AE9a leukemic mouse were injected into the tail veins of sublethally irradiated recipient mice (4.5 Gy).

Genomic PCR

Genomic DNA was isolated from tissues and cells using the Proteinase K DNA extraction method. Allele-in-one mouse tail lysis buffer (Fisher Scientific, #NC0504374) was utilized to extract

genomic DNA from mouse tails. PCR was performed using KOD Hot Start DNA polymerase (EMD Millipore, #71086) with the following primers:

P1: 5'-CCTAAAGAAGAGGCTGTGCTTTGG-3'

P2: 5'-CATCAAGGAAACCCTGGACTACTG-3'

P3: 5'-TGGCAAAGAATTCCTCGAGTTAA-3',

P4: 5'-CGGACCGCTATCAGGACATA-3'

P5: 5'-TAGTCAGGCACGTCGTATGG-3'

LK cell enrichment

For experiments where $Lin^{-}cKit^{+}$ (LK) bone marrow cells were utilized, two enrichment steps were performed after the isolation of total bone marrow. Lin^{-} cells were first enriched using the mouse Lineage Depletion Kit (Miltenyi Biotec, #130-090-858) according to the manufacturer's instructions. $cKit^{+}$ cells were then enriched using mouse CD117 microbeads (Miltenyi Biotec, #130-091-224) according to the manufacturer's instructions.

Western blot analysis

Cells were isolated from bone marrow, spleen, liver, and kidney tissues, lysed by resuspension in 2x SDS sample buffer, and boiled for 15 minutes at 95°C. Blots were incubated with a 1:1,000 dilution of RUNX1 /AML1 (D33G6) XP® Rabbit mAb #4336 (Cell Signaling Technology) or a 1:500,000 dilution of β-Actin clone AC-15 mouse monoclonal antibody (Millipore Sigma, #A1978). Blots were then incubated with the following secondary antibodies according to the manufacturer's recommended dilutions: IRDye 800CW goat anti-rabbit IgG (LI-COR, #926-32211), IRDye 800CW goat anti-mouse IgG (LI-COR, # 926-32210), and IRDye 680RD goat anti-mouse IgG (LI-COR, #926-68070). Immunoblotting was performed using the LI-COR Odyssey Class Infrared Imagine System

(Lincoln, Nebraska). Image quantitation was performed using LI-COR Image Studio Lite software (V 5.2.5).

Flow cytometry

Flow cytometric analyses were performed on a BD FACSCanto or BDFACSAriaII using the BD FACSDiva acquisition software. Post-acquisition data analysis was performed using the FlowJo software (FlowJo, LLC). The antibodies utilized were as follows:

PE anti-mouse CD3 (Biolegend, #100308)

PE anti-mouse CD11b (Biolegend, #101218)

PE anti-mouse Gr-1 (Biolegend, #108408)

PE anti-mouse CD16/CD32 (eBioscience, #12-0161-83)

PE anti-mouse CD48 (Biolegend, #103406)

PE anti-mouse CD34 (BD Pharmingen, #551387)

PerCP/Cy5.5 anti-mouse CD3ε (Biolegend, #100328)

PerCP/Cy5.5 anti-mouse CD4 (Biolegend, #100540)

PerCP/Cy5.5 anti-mouse CD8α (eBioscience, #45-0081-82)

PerCP/Cy5.5 anti-mouse CD11b (Biolegend, #101228)

PerCP/Cy5.5 anti-mouse Gr-1 (Biolegend, #1084280)

PerCP/Cy5.5 anti-mouse Ter-119 (Biolegend, #116228)

PerCP/Cy5.5 anti-mouse B220 (Biolegend, #103236)

PerCP/Cy5.5 anti-mouse CD19 (eBioscience, #45-0193-82)

PerCP/Cy5.5 anti-mouse CD127 (eBioscience, #45-1271-82)

PE/Cy7 anti-mouse CD117 (Biolegend, #105814)

PE/Cy7 anti-mouse Sca-1 (Biolegend, #122514)

APC anti-mouse CD117 (Biolegend, #105812)

APC anti-mouse CD3ε (eBioscience, #17-0031-82)

APC anti-mouse CD11b (Biolegend, #101212)

APC anti-mouse B220 (Biolegend, #103212)

APC anti-mouse Sca-1 (Biolegend, #108112)

Biotin anti-mouse CD150 (eBioscience, #13-1501-82)

Biotin anti-mouse CD34 (eBioscience, #13-0341-82)

APC/AlexaFlour750 Streptavidin (Molecular Probes, #SA1027)

Brilliant Violet 421™ anti-mouse CD135 Antibody (Biolegend, #135315)

Tissue fixation and histology

Spleens and livers were fixed with 4% paraformaldehyde (Fisher Scientific, #BP531-500) in PBS overnight at room temperature and then stored at 4°C. Paraffin-embedded sections were cut to a 5 μm thickness and stained with hematoxylin and eosin. Tissue processing and staining were performed by the UCSD Moores Cancer Center Tissue Technology Shared Resource.

Wright-Giemsa staining

To examine cell morphology, cytospins were prepared using the Cytopro 7620 Cytocentrifuge (Wescor). 100 µL of bone marrow or spleen cells (~2 x 10^6/mL) were added to each cytospin chamber, glass slides were loaded into the appropriate slots, and the samples were centrifuged at 500rpm for 5 minutes. Slides were fixed in 100% methanol for 30-40 seconds then stained with Wright solution (Sigma, WS16) followed by a PBS wash. Next, slides were stained in Giemsa solution (Sigma, GS500) diluted 1:10 in sodium phosphate buffer (pH 6.4) supplemented with 10% Triton-X. Slides were rinsed with water, dried overnight, and mounted. Images were acquired using an Olympus BX51 microscope equipped with a DP71 digital camera using the DP-BSW acquisition software (Olympus Corporation; Shinjuku, Tokyo, Japan).

Differential cell counts of murine bone marrow were performed on Wright-Giemsa-stained slides. For each replicate, 200 cells were counted.

Peripheral blood analysis

Peripheral blood samples were collected in EDTA-coated microvettes (Fisher Scientific, #NC9299309) by submandibular venipuncture with 5-mm animal lancets (Braintree Scientific, #GR5MM). Blood parameter measurements were performed using a Scil Vet abc Plus+ instrument (Henry Schein Animal Health).

Colony forming unit and serial replating assay

LK cells were isolated from the bone marrow of R26-WT and R26-AE9a as described. 5,000 LK cells were seeded in duplicate in 1 mL of MethoCult GF M3434 (StemCell Technologies, #03434) per 35-mm plate. After 7 days, the number of colonies and total number of cells per plate were recorded. 40,000 cells were then replated in duplicate in 1 mL of MethoCult GF M3434 per 35-mm plate. Colony analysis was repeated every 7 days for the duration of the experiment.

Colony imaging and area calculation

Colonies were identified by phase-contrast microscopy using a Zeiss Axiovert microscope and imaged with an attached camera. Colony area was calculated using ImageJ analysis software. Images were first converted to 8-bit grayscale, thresholded by signal, and masked to separate colonies from the background. A lower size limit was applied to filter signal noise and individual cells from colonies. Colony area was calculated by drawing polygonal outlines adhering to each threshholded colony in each image. Signal was quantified by using ImageJ's "measure" function. Partial images of colonies were excluded from area calculations.

Kasumi-1 AE-FKBP RNA-seq

Cell line

Kasumi-1 AE-FKBP cells were maintained in RPMI-1640 supplemented with 10% Fetal Bovine Serum, 100 U/ml penicillin, and 100 µg/ml streptomycin at 37°C and 5% CO_2. Inducible degradation of AML1-ETO was performed by adding 50 nM of dTAG^V-1 (Biotechne/Tocris Bio) directly to Kasumi-1 AE-FKBP culture media. The cell line was not recently authenticated or tested for mycoplasma.

RNA-seq library preparation

Kasumi-1 AE-FKBP cells were treated with either DMSO (control) or 50 nM dTAG^V-1 in 3 biological replicates for 12 hours to induce robust AE degradation. Total RNA was isolated from treated cells via Trizol (Invitrogen) extraction, following the manufacturer's protocol. Purified RNA was submitted to Novogene (Sacramento, CA) for poly-A enrichment-based library preparation and sequencing on the Illumina Novaseq (Paired End 150) Next Generation Sequencing platform.

Data processing and differential gene expression analysis

Pre-processed reads were aligned to the human genome (hg38.p13) using Bowtie2 (v2.4.5) and converted to BAM files using Samtools (v1.10). Picard MarkDuplicates (v2.26.10) was used to mark and remove sequence duplicates in processed BAM files. FeatureCounts (v2.0.0) and the corresponding Refseq hg38.p13 genomic annotation were used to quantify raw counts. Differentially expressed genes were identified using DESeq2[2] comparing dTAG^{V}-1 treated cells to DMSO treated control cells using the default settings.

Single-cell RNA sequencing (scRNA-seq) data generation and data processing

10x genomics library preparation

LK cells were enriched as previously described. Single-cell suspensions were converted to barcoded libraries using the 10X Genomics Chromium Single Cell 3' GEM, Library & Gel bead Kit V3 and tagged using the Chromium i7 Multiplex Kit. Libraries were sequenced using Illumina Next Generation Sequencing systems (HiSeq4000).

Data Processing, quality control, and dataset integration

Cell ranger output matrices (feature, count, barcode) for both AE9a and Control samples were processed using Seurat V3 (version 3.1.5)[3]. Cells with UMI count less than 500, gene count less than 250, and mitochondrial genome transcripts greater than 10% were removed. Genes expressed in less than 10 cells were removed. Post filtering, the control dataset consisted of 5896 cells and the AE9a dataset consisted of 6511 cells. Each dataset was normalized using the "NormalizeData" function with default arguments. The top 2000 variable features were identified using "FindVariableFeatures" with the "vst" selection method. To permit comparative analysis between AE9a and Control RNA-seq datasets, both samples were integrated using Seurat's "FindIntegrationAnchors" and "IntegrateData" functions using the default arguments. The "ScaleData"

function was utilized with the default arguments to center and scale the new dataset. After clustering and marker gene identification, we chose to subset the dataset to exclude cells belonging to 4 clusters deemed to be unrelated to our study. The resultant subset was re-clustered before continuing with downstream analysis.

Cell clustering, dimensional analysis, and visualizations

Cell clustering and dimensional analysis were performed using the Seurat package (version 3.1.5)[3]. After integration, we performed a Principal Component Analysis (PCA) using “RunPCA”. We attempted to reduce technical noise by selecting only significant Principal Components as determined by elbow plot and repeated analysis with different numbers of principal components. We chose to use the first 30 principal components as our standard for downstream analyses. Cells were clustered using the “FindClusters” function using the Louvain algorithm. We set the “resolution” parameter to 0.5 to identify only major clusters. Uniform Manifold Approximation and Projection (UMAP) was used to graph the clusters into two-dimensional space using “RunUMAP” and visualized using the “DimPlot” function. The “split.by” and “group.by” arguments in the “DimPlot” function were used to visually distinguish AE9a and Control cells where appropriate. Marker genes for clusters were visualized using “FeaturePlot”.

Subclustering analysis was performed using standard Seurat functions. Briefly, clusters of interest from the initial integration were subset using the Seurat “subset” function to generate new Seurat datasets. New datasets were then re-processed using “ScaleData”, “RunPCA”, “RunUMAP”, “FindNeighbors”, and “FindClusters” to recluster cells and generate new subclusters using default and developer recommended settings. Top marker genes for new subclusters were identified using the “FindAllMarkers” function and visualized using the “DoHeatmap” function.

Differential gene expression analysis

MAST (version 1.12.0)[4] in Seurat was used for initial cluster marker gene identification ("FindAllMarkers") and for all differential gene expression analyses ("FindMarkers") between clusters and/or conditions using the default arguments. For each cluster, differentially expressed genes (DEGs) were identified relative to all other clusters. Additionally, for each cluster, DEG lists were generated between AE9a and Control cells.

Pseudobulk analysis was performed using the DElegate[5] package. Briefly, single cells were assigned to subgroups using the Seurat "subset" function based on metadata qualifiers such as AE9a/Control or cluster number. Pseudobulk groups were generated from subsets using DElegate with developer recommended settings, and DEseq2 was used to perform differential gene expression analysis between the indicated groups and obtain lists of DEGs for downstream analyses.

Unbiased cell type annotation

Cell type annotation was performed using cellHarmony (AltAnalyze version 2.1.3)[6] and examination of cluster marker genes. cellHarmony is a computational workflow within the AltAnalyze analysis suite that attempts to assign cell types to a query dataset by similarity to a reference set of cell types. To assign cell types to our data, we first generated a reference dataset using published scRNA-seq data from Nestorowa et al. 2016[7]. Next, we used this reference with cellHarmony to assign cell type labels to the integrated dataset. Subsequent analyses were conducted using both the unsupervised labeling as well as specific cluster numbers where appropriate.

Pseudotime /trajectory inference

Pseudotime analysis was performed with STREAM (version 0.3.9)[8]. The top 2000 highly variable features in Control scRNA-seq data were used to construct multi-furcated differentiation trajectories according to STREAM vignettes located on the GitHub repository. The root node of the

trajectory was selected based on which cluster was assigned most immature (HSC/MPP) by unsupervised cellHarmony analysis. Mapping of AE9a scRNA-seq data onto the control cell trajectory was done with cells belonging to specific AE9a clusters as described in STREAM vignettes.

Regulatory Network Analysis (Regulon/SCENIC)

A single-cell regulatory network analysis was performed on all control and AE9a cells using SCENIC[9]. Briefly, scRNA-seq data was analyzed for co-expression patterns correlated with transcription factors to create predicted regulatory networks. To create regulons, network genes were queried for enrichment of regulatory TF DNA binding motifs in their TSS regions where genes without significant enrichment were pruned from the network. Finally, regulon activity was scored by enrichment of regulon associated gene expression across all genes in a particular cell. Both positive and negative regulons can be identified by corresponding positive or negative correlation of regulated gene expression with a particular transcription factor in the regulon creation step. We specifically used pySCENIC (version 0.10.0) with implemented GRNBoost2 and cisTarget functions to infer regulatory networks and discover transcription factor motifs. Analyses were performed using the motifs-v9-nr.mgi-m0.001-o0.0 database and mm9 mc9r 500bpUp and TSS+/-10kb motif ranking lists (https://resources.aertslab.org/cistarget/). Regulon activity in individual cells was scored using AUCell (version 1.8.0)[9]. Heatmaps of regulon-cell activity were generated using the pheatmap and ComplexHeatmap R packages. Regulon activity scores were scaled by row Z-score before plotting.

AUCell

To score control or AE9a cell clusters as more or less GMP or MEP like, we first identified the top 50% of expressed genes by Gini coefficient. Next, we defined custom gene sets containing highly expressed genes in GMPs or MEPs from a published scRNA-seq dataset[10]. Using AUCell, we then calculated an area-under-curve (AUC) enrichment score for the custom gene sets in the clusters of

interest for both AE9a and Control samples. The distribution of scores were compared by Student's T-test with significantly different AUC score distributions having P-value < 0.05.

Functional enrichment analyses

Gene set enrichment analysis (GSEA)[11, 12] was performed using the MSigDB C2 collection on pre-ranked lists of DEGs (R26-AE9a vs. R26-WT) generated from Seurat MAST DEG analysis.

Gene Ontology and gene-disease association analyses were performed using Metascape[13, 14] with default analysis settings.

Statistical analyses

All statistical analyses were performed using the GraphPad Prism software (Version 9.1.0). We assumed that groups being statistically compared had similar variances and were from at least three biological replicates to ensure adequate power. The one exception to this is comparison of colony cell number in Figure 4B where two biological replicates each with two technical replicates were compared. All statistical tests, sample sizes, and number of experimental replicates are documented in the respective figure legends. Figure legends also contain information regarding the center value and error bars displayed for each experiment. P values are denoted as follows: * $p < 0.05$, ** $p < 0.01$, and *** $p < 0.001$.

Data availability

The scRNA-seq data has been uploaded to the Gene Expression Omnibus (GEO) under accession GSE173712. The DNA construct used in this study will be made available upon email request to the corresponding author.

SUPPLEMENTARY REFERENCES

1. Yan, M., et al., *A previously unidentified alternatively spliced isoform of t(8;21) transcript promotes leukemogenesis.* Nat Med, 2006. **12**(8): p. 945-9.
2. Love, M.I., W. Huber, and S. Anders, *Moderated estimation of fold change and dispersion for RNA-seq data with DESeq2.* Genome Biology, 2014. **15**(12): p. 1-21.
3. Stuart, T., et al., *Comprehensive Integration of Single-Cell Data.* Cell, 2019. **177**(7): p. 1888-1902.e21.
4. Finak, G., et al., *MAST: a flexible statistical framework for assessing transcriptional changes and characterizing heterogeneity in single-cell RNA sequencing data.* Genome Biol, 2015. **16**: p. 278.
5. Hafemeister, C. and F. Halbritter, *Single-cell RNA-seq differential expression tests within a sample should use pseudo-bulk data of pseudo-replicates.* biorXiv, 2023.
6. DePasquale, E.A.K., et al., *cellHarmony: cell-level matching and holistic comparison of single-cell transcriptomes.* Nucleic Acids Res, 2019. **47**(21): p. e138.
7. Nestorowa, S., et al., *A single-cell resolution map of mouse hematopoietic stem and progenitor cell differentiation.* Blood, 2016. **128**(8): p. e20-31.
8. Chen, H., et al., *Single-cell trajectories reconstruction, exploration and mapping of omics data with STREAM.* Nat Commun, 2019. **10**(1): p. 1903.
9. Aibar, S., et al., *SCENIC: single-cell regulatory network inference and clustering.* Nat Methods, 2017. **14**(11): p. 1083-1086.
10. Laurenti, E., et al., *The transcriptional architecture of early human hematopoiesis identifies multilevel control of lymphoid commitment.* Nat Immunol, 2013. **14**(7): p. 756-63.
11. Mootha, V.K., et al., *PGC-1alpha-responsive genes involved in oxidative phosphorylation are coordinately downregulated in human diabetes.* Nat Genet, 2003. **34**(3): p. 267-73.
12. Subramanian, A., et al., *Gene set enrichment analysis: a knowledge-based approach for interpreting genome-wide expression profiles.* Proc Natl Acad Sci U S A, 2005. **102**(43): p. 15545-50.
13. Y, Z., et al., *Metascape provides a biologist-oriented resource for the analysis of systems-level datasets.* Nature communications, 2019. **10**(1).
14. J, P., et al., *The DisGeNET knowledge platform for disease genomics: 2019 update.* Nucleic acids research, 2020. **48**(D1).
15. Piovan, C., et al., *Generation of mouse lines conditionally over-expressing microRNA using the Rosa26-Lox-Stop-Lox system.* Methods Mol Biol, 2014. **1194**: p. 203-24.

SUPPLEMENTARY TABLES

SUPPLEMENTARY TABLE 1. Composition of bone marrow in R26-AE9a leukemic mice and R26-WT control mice.

Bone marrow was harvested from R26-WT and R26-AE9a leukemic mice and cytospun onto slides. Following Wright-Giemsa staining, differential counts were performed under a microscope. Data are mean ± SD. In each bone marrow sample, 200 cells were counted.

	Differential Counts, %						
**Mouse Genotype**	Myeloblasts / Promyelocytes	Metamyelocytes / Myelocytes	Lymphoid	Neutrophils	Eosinophils	Monocytes	Erythroid
**R26-WT Control (n = 2)**	7.5 ± 1.4	14 ± 2.8	15 ± 1.4	43.3 ± 3.9	1.3 ± 0.4	5 ± 2.8	14 ± 2.2
**R26-AE9a Leukemic (n = 2)**	20.3 ± 2.5	20 ± 2.8	5 ± 2.8	35 ± 4.2	3.5 ± 0.7	8 ± 2.1	8.3 ± 1.1

SUPPLEMENTARY FIGURE LEGENDS

SUPPLEMENTARY FIGURE 1. Generation of Rosa26-AE9a KI mice.

A. PCR analysis of genomic DNA extracted from various embryonic stem cell clones. Primers P4/P5 (Figure 1A) were utilized to confirm integration of the transgene into the R26 locus. Positive control DNA was derived from transgenic mice described in Piovan et al.[15]

B. Western blot analysis of AE9a (anti-RUNX1 antibody) and β-actin protein (loading control) in hematopoietic and non-hematopoietic tissues of the indicated mice.

C. Representative flow cytometry histograms of GFP^+ cells in the bone marrow (BM), spleen (SP), and peripheral blood (PB) of R26-WT versus R26-AE9a mice. The data for R26-AE9a mice are summarized in Figure 1D.

SUPPLEMENTARY FIGURE 2. Rosa26-AE9a mice develop AML.

A. Representative flow cytometry charts of cKit expression in the bone marrow (BM) of a leukemic R26-AE9a mouse. These data are summarized by the bar graph in Figure 2B.

B. Representative flow cytometry charts of Cd34 expression in the BM of a leukemic R26-AE9a mouse. These data are summarized by the bar graph in Figure 2C.

C. Representative flow cytometry charts of Cd11b, Gr-1, Cd3, and B220 in the BM of a leukemic R26-AE9a mice. These data are summarized by the bar graphs in Figure 2D.

D. Hematoxylin and eosin (H&E) staining of liver sections taken from a representative R26-WT mouse and R26-AE9a leukemic mouse. Tissue was collected from the leukemic mice when they were deemed moribund. Age-matched R26-WT mice serve as the control.

E. Western blot analysis of AE9a (anti-RUNX1 antibody) and β-actin protein (loading control) in BM of the indicated R26-WT or R26-AE9a transgenic mice (lanes 1-5), wild type BM infected with the MSCV-IRES-puromycin AE9a retroviral overexpression construct prior to transplantation (lanes 6-7), and BM of a leukemic mouse following bone marrow

transplantation (BMT) of the AE9a-transduced cells (lane 9). Quantifications of AE9a protein relative to β-actin are shown below each lane.

SUPPLEMENTARY FIGURE 3. Transplantation of Rosa26-AE9a BM increases leukemia penetrance.

A. Schematic depicting the transplantation of BM cells from R26-AE9a mice into irradiated recipient mice.
B. Kaplan-Meier survival curve depicting the proportion of mice transplanted with R26-AE9a BM that developed leukemia over the course of 18 months. n = 8 for both R26-WT BMT and R26-AE9a BMT. P-value is shown; one-sided Log-rank (Mantel-Cox) test.
C. Flow cytometric analysis of the percentage of cKit$^+$ cells in the BM and SP of one of the mice transplanted with R26-AE9a BM that developed leukemia. Data were collected when the leukemic mouse was deemed moribund and are representative of both transplanted mice that developed leukemia.
D. Wright-giemsa staining of BM and spleen cytospins from a representative leukemic mouse transplanted with R26-AE9a BM.

SUPPLEMENTARY FIGURE 4. Representative FACS of progenitor cell compartments.

A. Flow cytometry charts depicting the analysis of Lin$^-$Sca-1$^+$cKit$^+$ (LSK) cells and various progenitor populations in the bone marrow (BM) of a representative R26-WT mouse at 12 weeks. These data are summarized by the bar graphs in Figure 3A-B. MPP: multipotent progenitor; HSC: hematopoietic stem cell.
B. Flow cytometry charts depicting the analysis of common myeloid progenitors (CMP), granulocyte-monocyte progenitors (GMP), and megakaryocyte-erythroid progenitors (MEP) in the BM of a representative R26-WT mouse at 12 weeks. These data are summarized by the bar graphs in Figure 3C.

SUPPLEMENTARY FIGURE 5. Mature blood cell populations are unchanged in Rosa26-AE9a mice.

A. Quantifications of various mature blood cell populations over time in the peripheral blood of R26-WT and non-leukemic R26-AE9a mice. For each parameter, data represents either the total cell number or the percentage of all nucleated cells in the peripheral blood (PB). WBC: white blood cells; RBC: red blood cells. Data are not significantly different at any timepoint using multiple Student's t-tests and the Holm-Sidak method to correct for multiple comparisons.

B/C. Flow cytometric analysis of the percentage of $Cd3^+$, $B220^+$, $Cd11b/Gr\text{-}1^+$, and $cKit^+$ cells in the bone marrow (B) and spleen (C) of R26-WT and R26-AE9a mice at 12 weeks. R26-WT: n = 3; R26-AE9a: n = 3. None of the pairs are statistically significant using a Student's t-test.

SUPPLEMENTARY FIGURE 6. R26-AE9a mice exhibit gene expression changes analogous to human t(8;21) AML.

A. Venn diagram comparing (left) significantly differentially expressed genes from Kasumi-1 AE-FKBP cells with and without degron-induced AE degradation (adjusted $p < 0.05$) with (right) significantly differentially expressed genes in pseudobulk R26-AE9a cells versus R26-WT cells (adjusted $p < 0.05$).

B. DisGeNET gene-disease association enrichment analysis of the 385 overlapping genes in panel A.

C. Gene ontology (GO) enrichment analysis of the 385 overlapping genes in panel A.

SUPPLEMENTARY FIGURE 7. Marker genes validate cellHarmony labels of hematopoietic populations.

Feature plots depicting the expression of (A) stem cell marker genes, (B) granulocyte-monocyte progenitor (GMP) marker genes, (C) megakaryocyte-erythroid progenitor (MEP) marker genes, and (D) monocyte-dendritic cell progenitor (MDP) marker genes across all sequenced LK cells (R26-WT and R26-AE9a), organized by Uniform Manifold Approximation and Projection (UMAP).

SUPPLEMENTARY FIGURE 8. Confirmation of the ordering of clusters along a hematopoietic differentiation trajectory.

A. Bar chart showing the expression of various erythroid differentiation genes across all R26-WT LK cells within the indicated clusters.
B. Pie graphs depicting the composition of cell types within each cluster of the indicated sample. HSC: hematopoietic stem cells; MPP: multipotent progenitors; CMP: common myeloid progenitors; GMP: granulocyte-monocyte progenitors; MDP: monocyte-dendritic cell progenitors; MEP: megakaryocyte-erythroid progenitors.
C. Bar chart showing the expression of various myeloid differentiation genes across all R26-WT LK cells within the indicated clusters.
D. Pie graphs depicting the composition of cell types within each cluster of the indicated sample.

SUPPLEMENTARY FIGURE 9. GMP lineage bias is observed in MEP-primed CMPs, but not HSPCs or GMP/MDP-primed CMPs.

AUCell analysis of the expression of both GMP and MEP gene sets in LK cells from R26-WT and R26-AE9a cluster 5 (A) and cluster 6 (B). No comparisons were significant by a Student’s t-test.

SUPPLEMENTARY FIGURE 10. R26-AE9a Cluster 0 cells acquire an immature granulocytic cellular identity.

A. (Left) Uniform Manifold Approximation and Projection (UMAP) of $Lin^{-}cKit^{+}$ (LK) cells isolated from the bone marrow (BM) of R26-WT (green) and R26-AE9a (blue) mice at 12 weeks. (Right) UMAP depicting subclusters of original Cluster 0.
B. Cluster 0 subclusters subdivided by mouse genotype: R26-WT (top) and R26-AE9a (bottom).
C. Heatmap depicting the ~15 top positive logFC marker genes for each subcluster.
D. Gene set enrichment analysis (GSEA) of subcluster 0 versus subclusters 2 and 3. NES: normalized enrichment score.
E. GSEA of subcluster 0 versus subcluster 1.

SUPPLEMENTARY FIGURE 11. R26-AE9a cluster 1 cells express a granulocytic gene signature.

A. (Left) Uniform Manifold Approximation and Projection (UMAP) of $Lin^{-}cKit^{+}$ (LK) cells isolated from the bone marrow (BM) of R26-WT (green) and R26-AE9a (blue) mice at 12 weeks. (Right) UMAP depicting subclusters of original Cluster 1.
B. Cluster 1 subclusters subdivided by mouse genotype: R26-WT (top) and R26-AE9a (bottom).
C. Heatmap depicting the ~15 top positive logFC marker genes for each subcluster.
D. Gene set enrichment analysis (GSEA) of subcluster 1 versus subclusters 0 and 2. NES: normalized enrichment score.

SUPPLEMENTARY FIGURE 12. Hematopoietic transcription factor activity reveals blocked differentiation in R26-AE9a mice.

A. Bar graph depicting normalized Cebpa expression across all cells in the indicated samples and clusters. Data are mean +/- s.d. *** $p < 0.001$; Kruskal-Wallis test with post-hoc uncorrected Dunn's test.
B. Heatmap depicting regulatory network (regulon) analysis of LK cells in the indicated clusters and samples. Row Z-scored AUCell enrichment scores for the Cebpe positive regulon within each cell are shown.
C. Violin plots quantifying the data depicted in (B). *** $p < 0.001$, **** $p < 0.0001$; Kruskal-Wallis test.
D. Bar graph depicting normalized Cebpe expression across all cells in the indicated samples and clusters. Data are mean +/- s.d. * $p < 0.05$; ** $p < 0.01$; Kruskal-Wallis test with post-hoc uncorrected Dunn's test.
E. Heatmap depicting regulatory network (regulon) analysis of LK cells in the indicated clusters and samples. Row Z-scored AUCell enrichment scores for Tal1 positive and negative regulons within each cell are shown.
F. Violin plots quantifying the data depicted in (E). **** $p < 0.0001$; Kruskal-Wallis test.
G. Bar graphs depicting normalized Gata1 and Tal1 expression across all cells in the indicated samples and clusters. Data are mean +/- s.d. None of the clusters had significantly different Gata1 or Tal1 expression in R26-WT versus R26-AE9a cells via a Kruskal-Wallis test with post-hoc uncorrected Dunn's test.

SUPPLEMENTARY FIGURE 13. Expression of Sox4 positive regulon genes in R26-WT and R26-AE9a mice.

A. Violin plots showing the distribution of Sox4 positive regulon associated genes expression across all cells within the indicated samples (R26-WT or R26-AE9a) and clusters (5, 6, 9, or 11).

Supplementary Figure 1. Generation of Rosa26-AE9a KI mice.

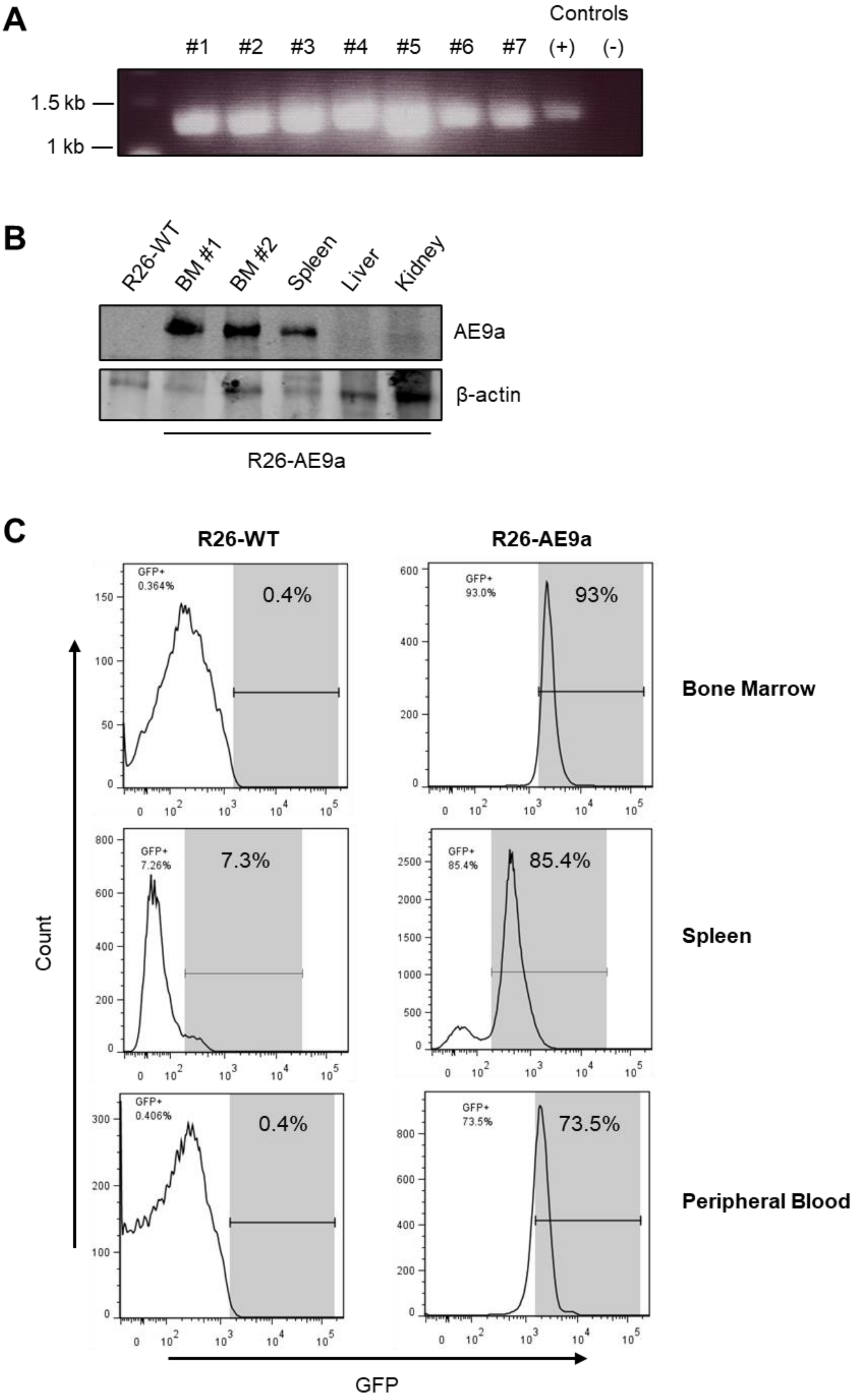

Supplementary Figure 2. Rosa26-AE9a mice develop AML.

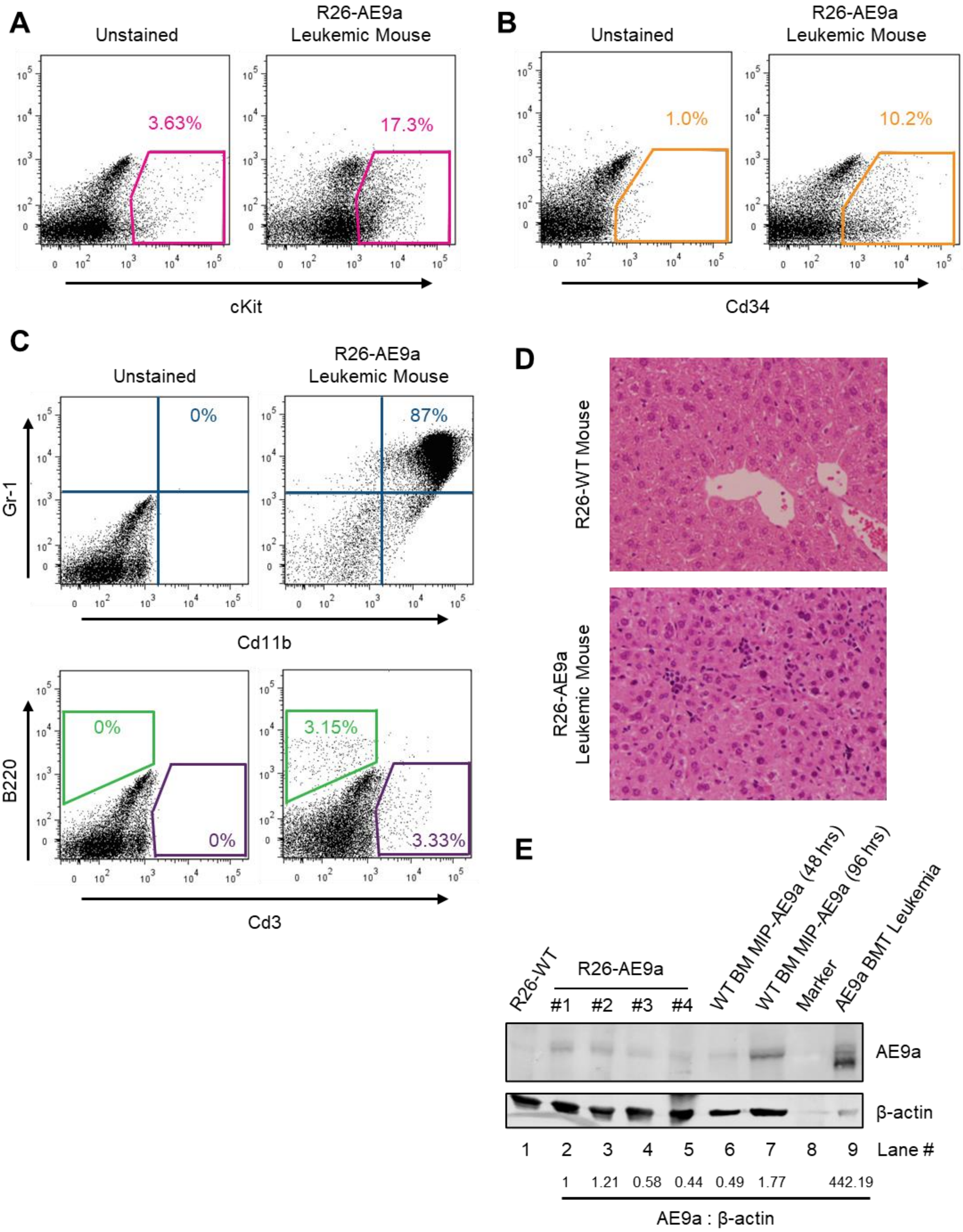

Supplementary Figure 3. Transplantation of Rosa26-AE9a BM increases leukemia penetrance.

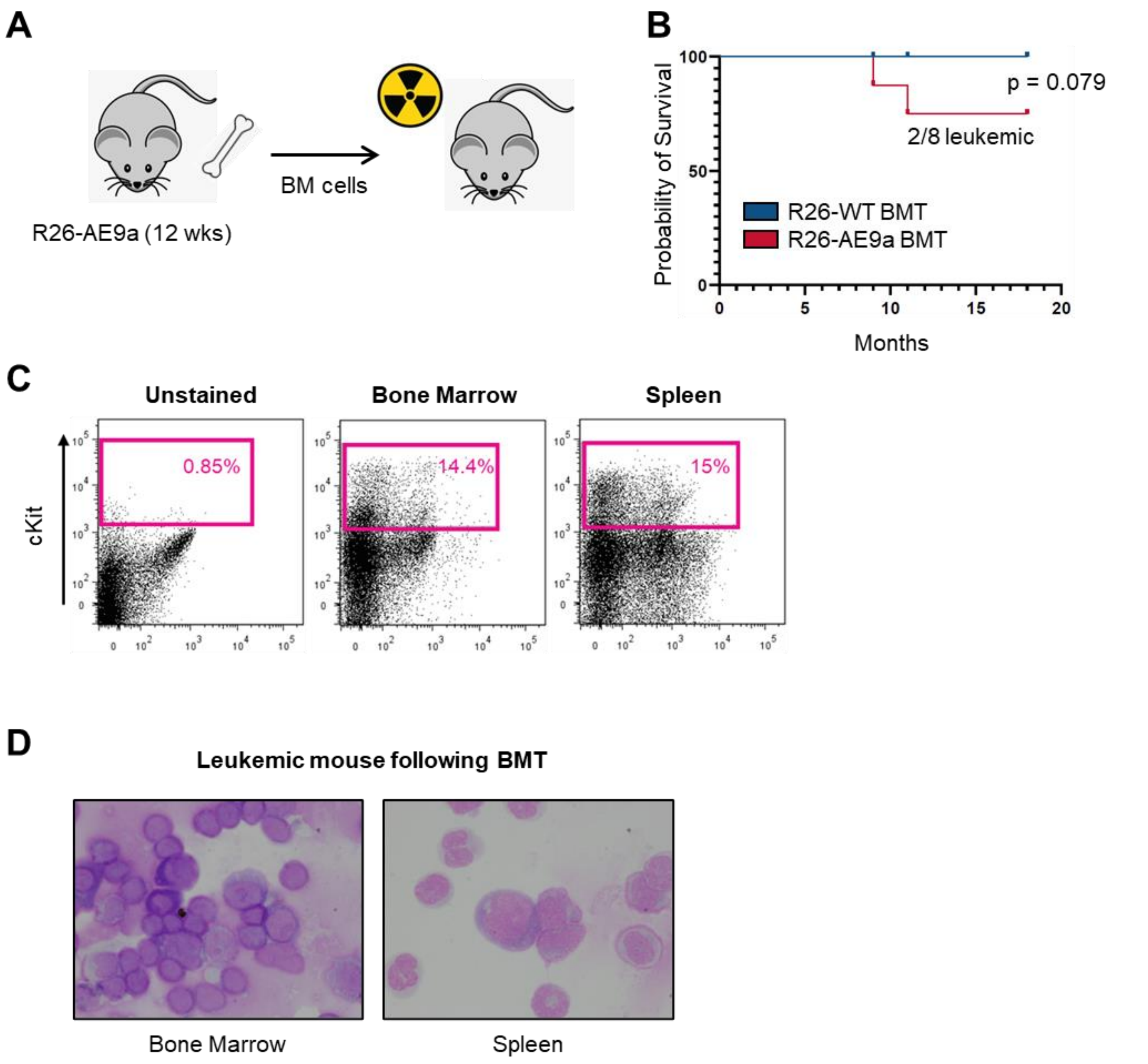

Supplementary Figure 4. Representative FACS of progenitor cell compartments.

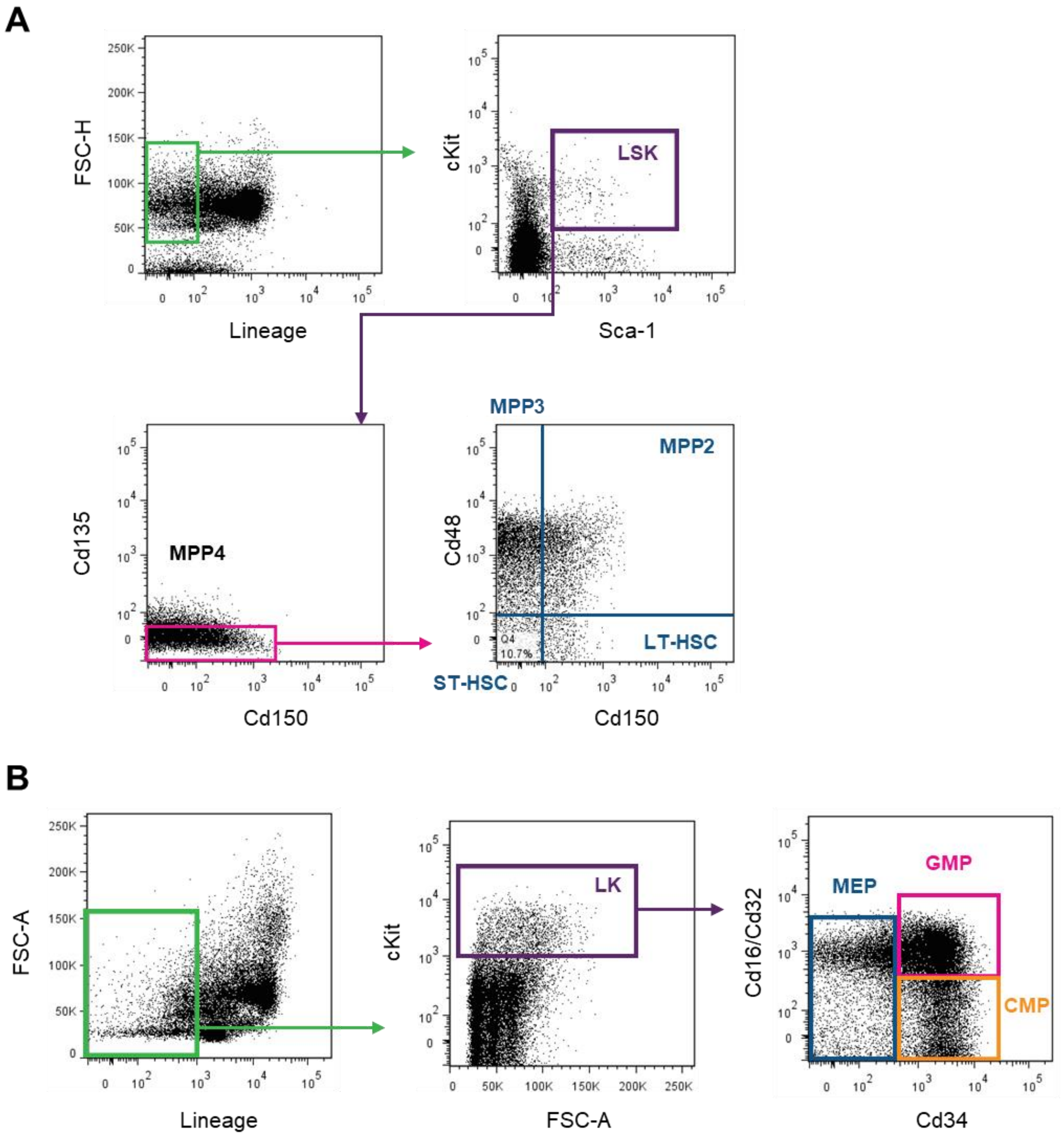

Supplementary Figure 5. Mature blood cell populations are unchanged in Rosa26-AE9a mice.

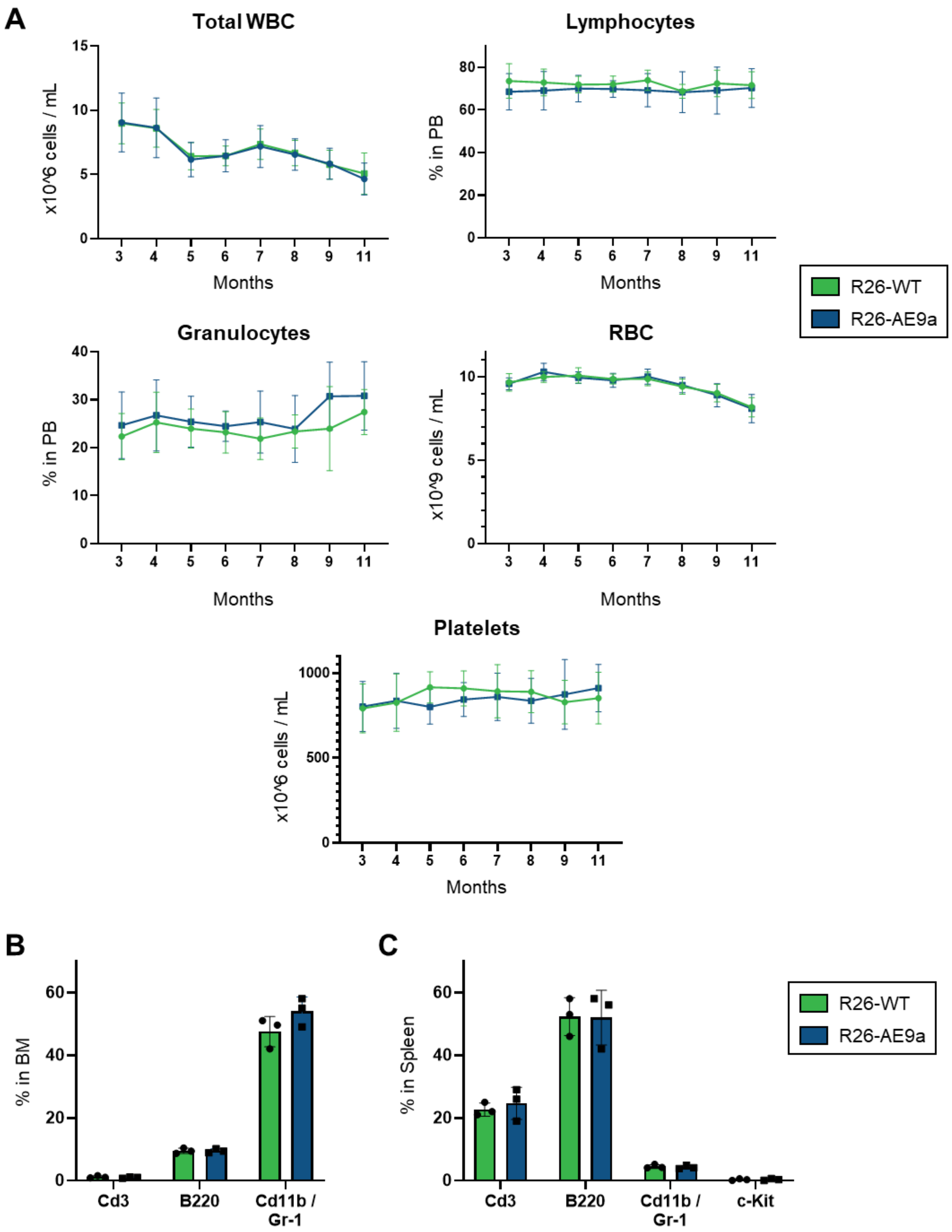

Supplementary Figure 6. R26-AE9a mice exhibit gene expression changes analogous to human t(8;21) AML.

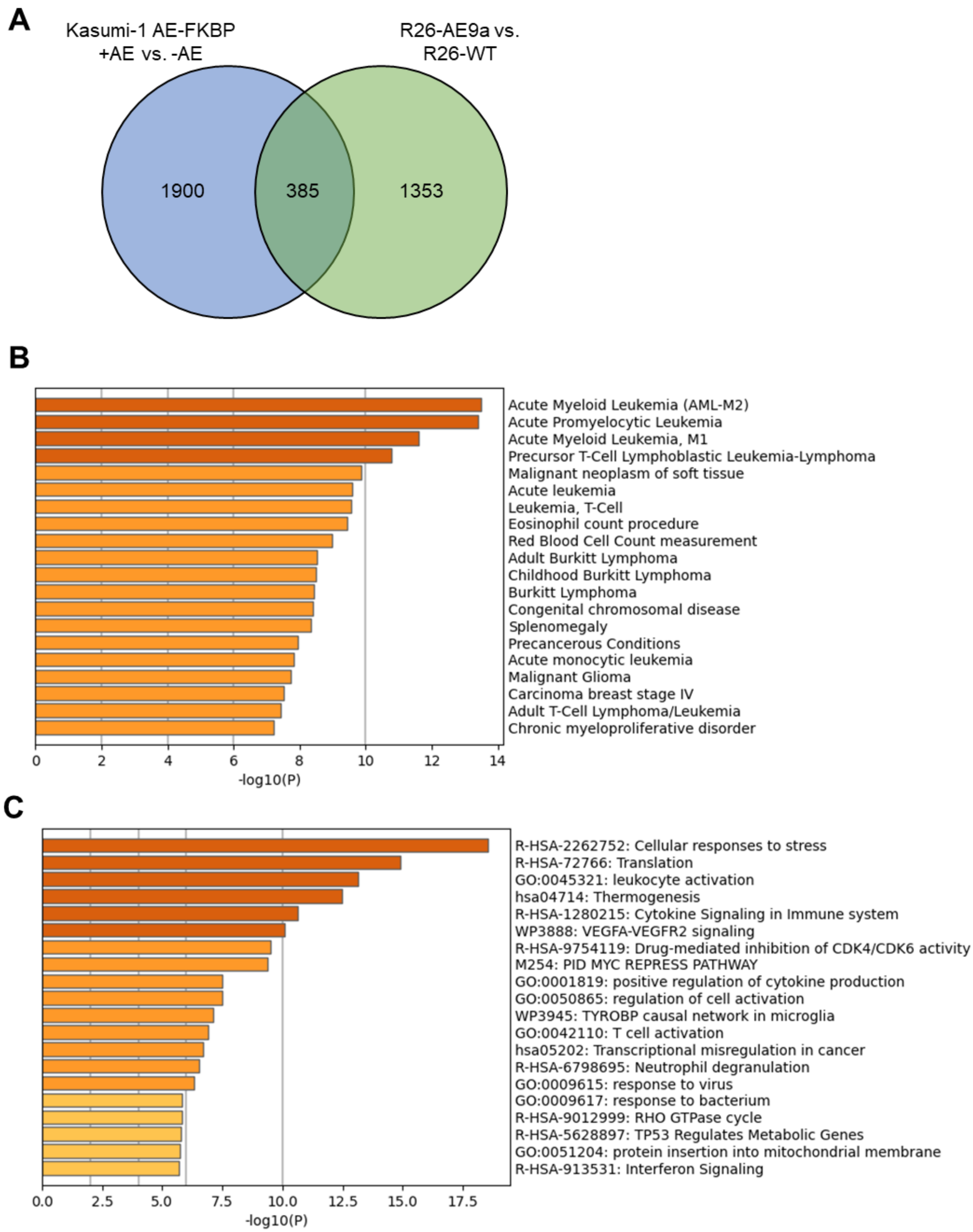

Supplementary Figure 7. Marker genes validate cellHarmony labels of hematopoietic populations.

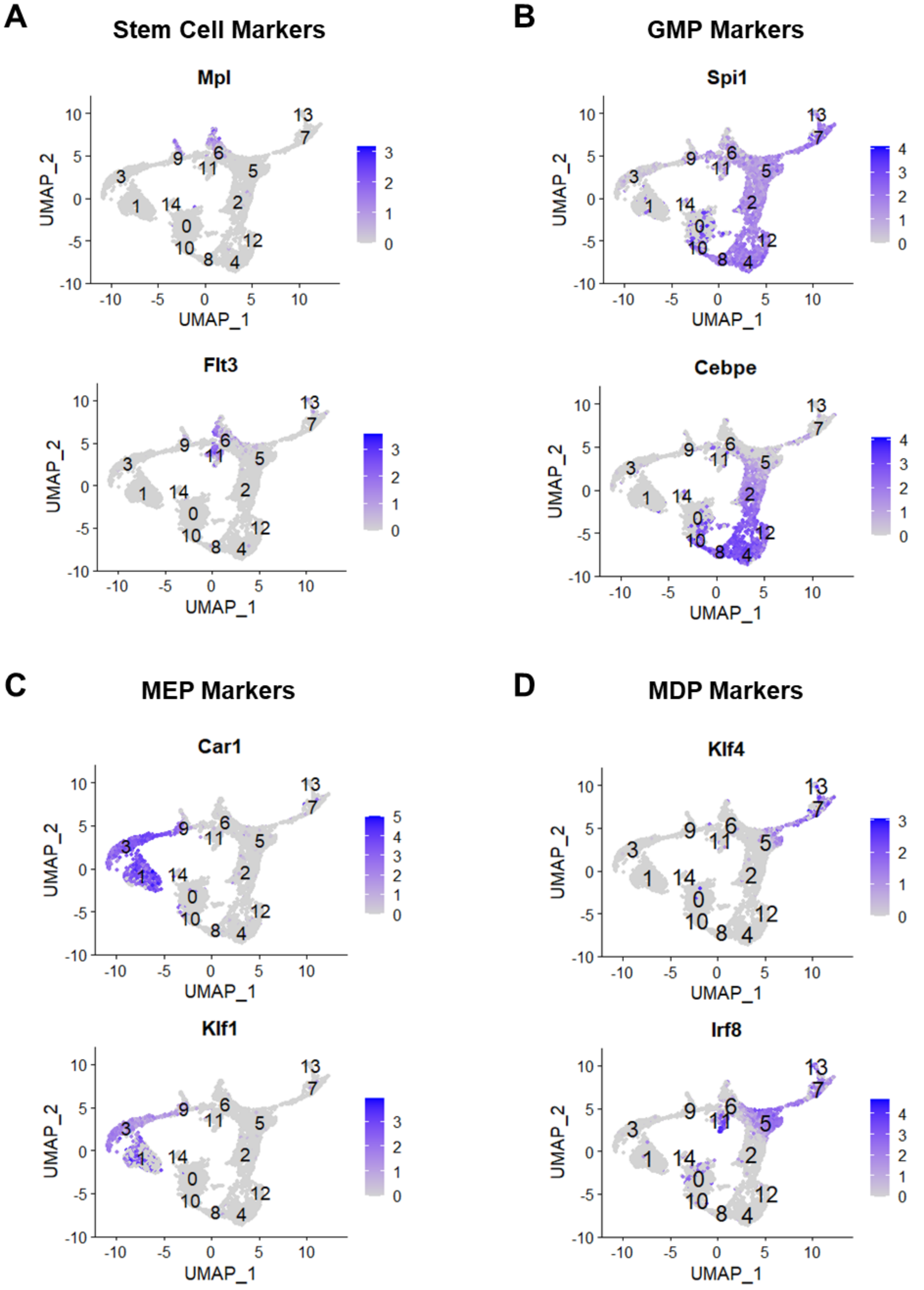

Supplementary Figure 8. Confirmation of the ordering of clusters along a hematopoietic differentiation trajectory.

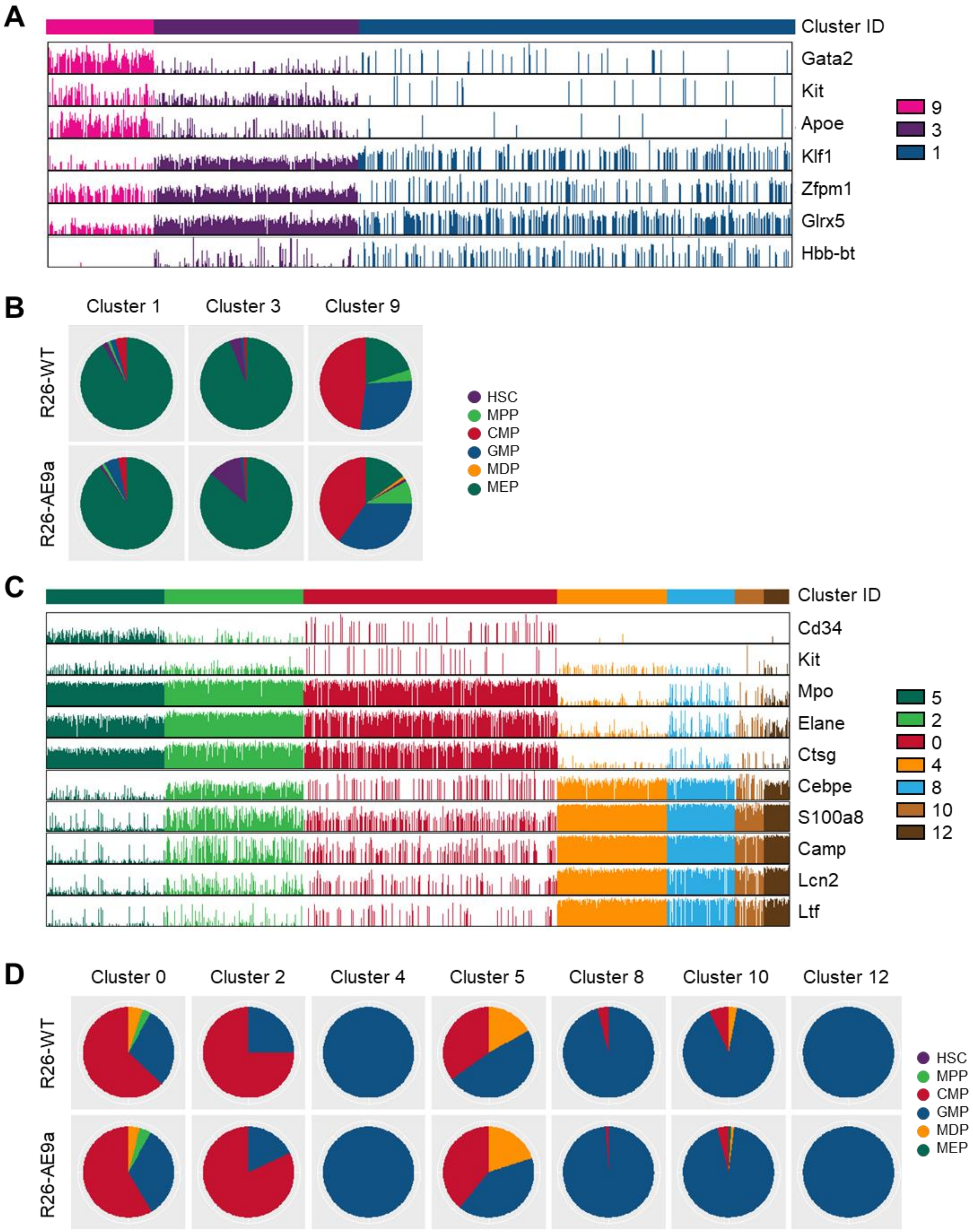

Supplementary Figure 9. GMP lineage bias is observed in MEP-primed CMPs, but not HSPCs or GMP/MDP-primed CMPs.

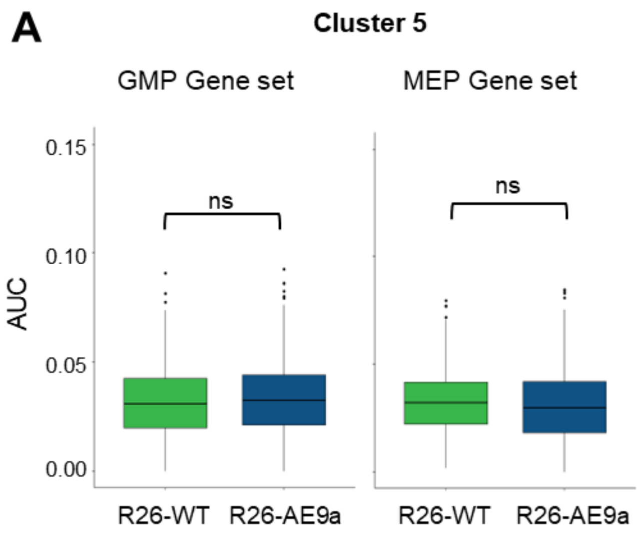

Supplementary Figure 10. R26-AE9a Cluster 0 cells acquire an immature granulocytic cellular identity.

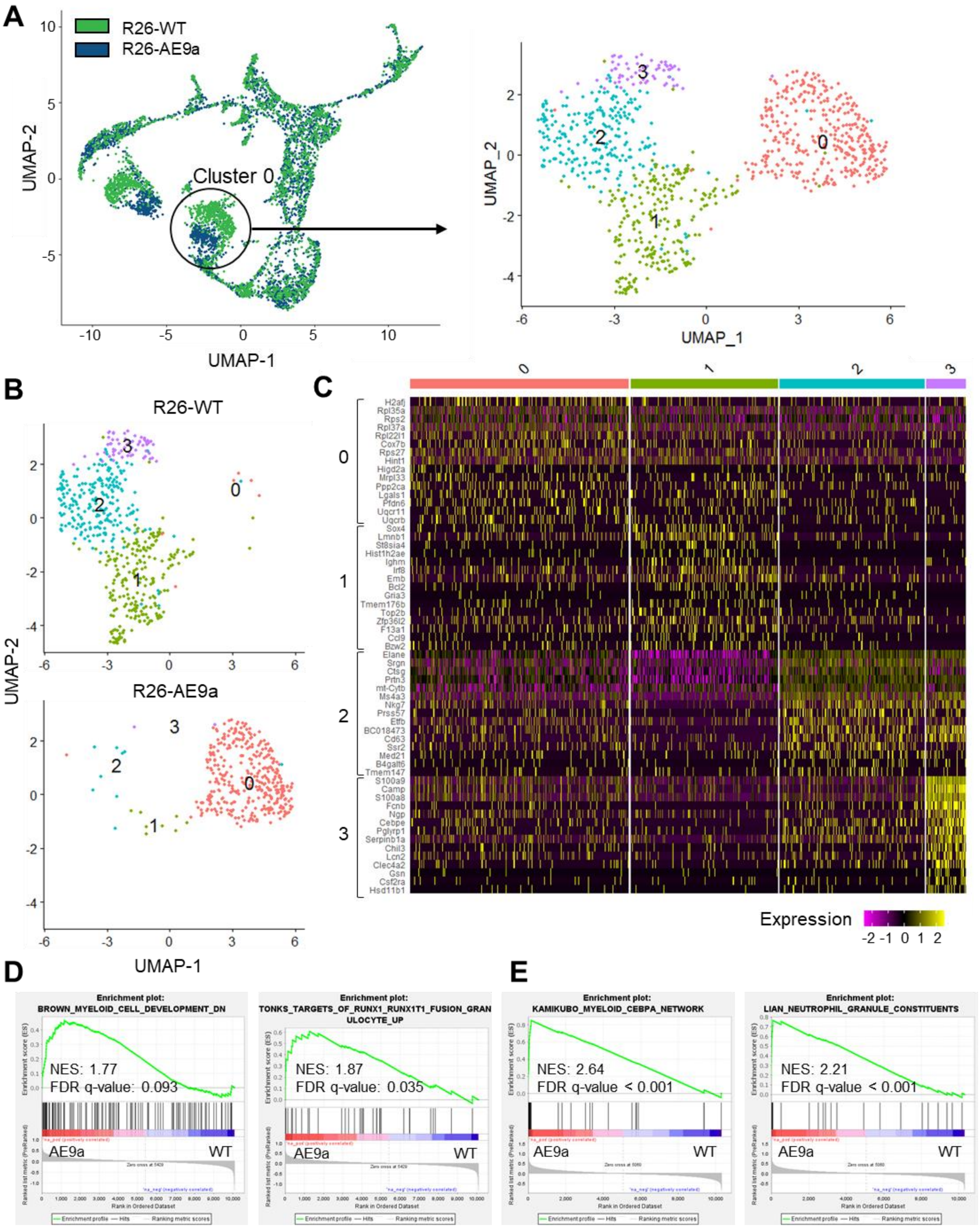

Supplementary Figure 11. R26-AE9a cluster 1 cells express a graulocytic gene signature.

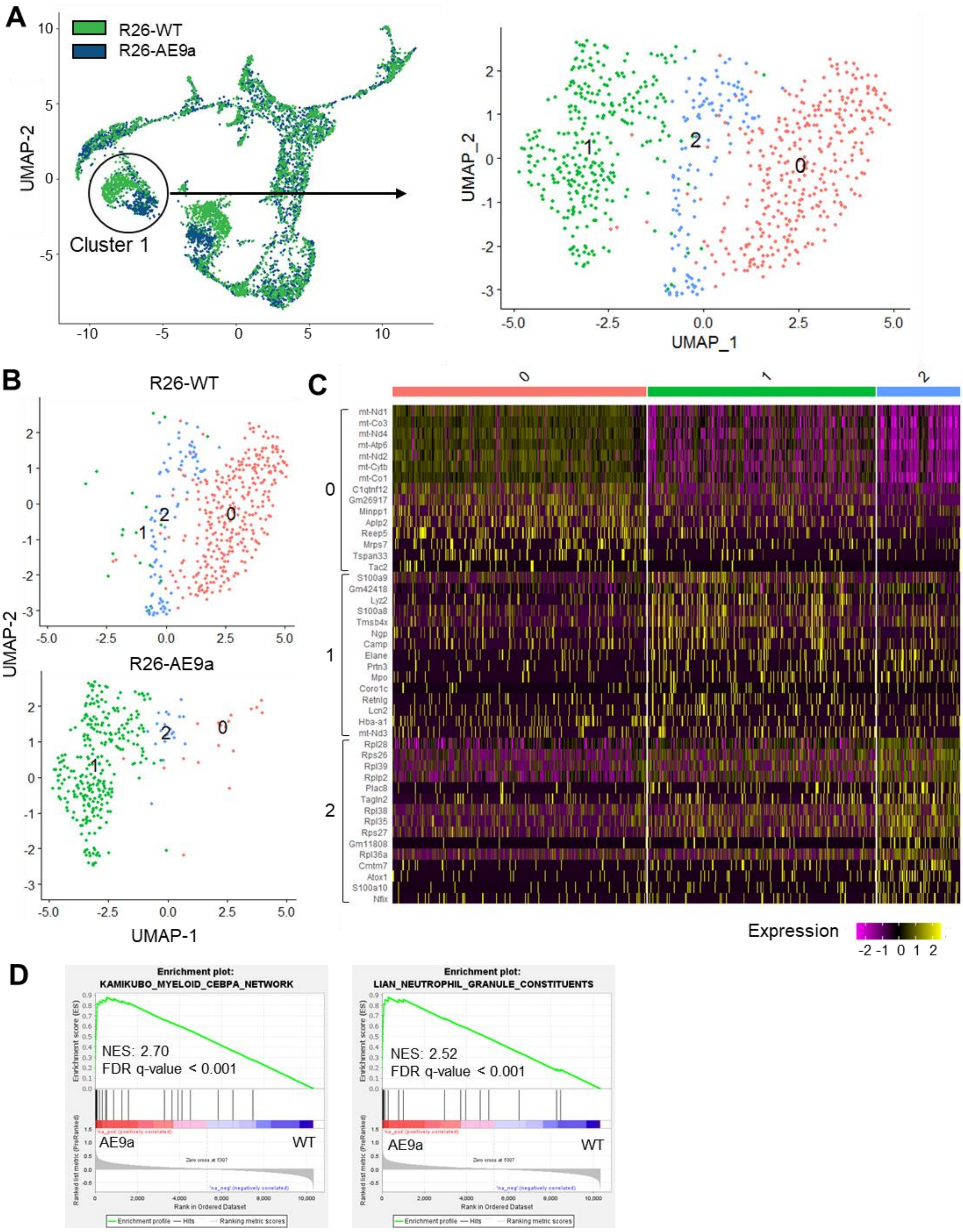

Supplementary Figure 12. Hematopoietic transcription factor activity reveals blocked differentiation in R26-AE9a mice.

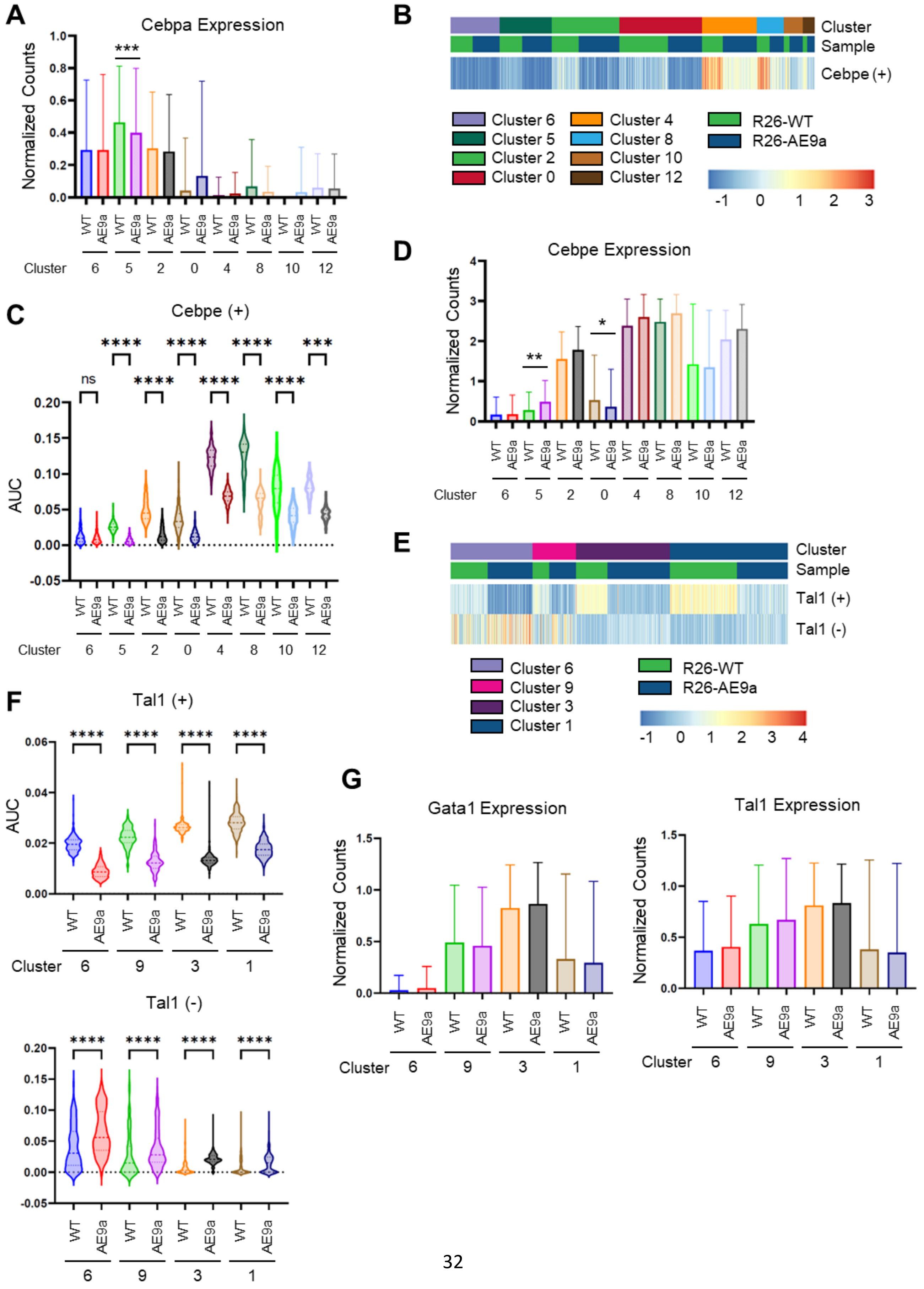

Supplementary Figure 13. Expression of Sox4 positive regulon genes in R26-WT and R26-AE9a mice.

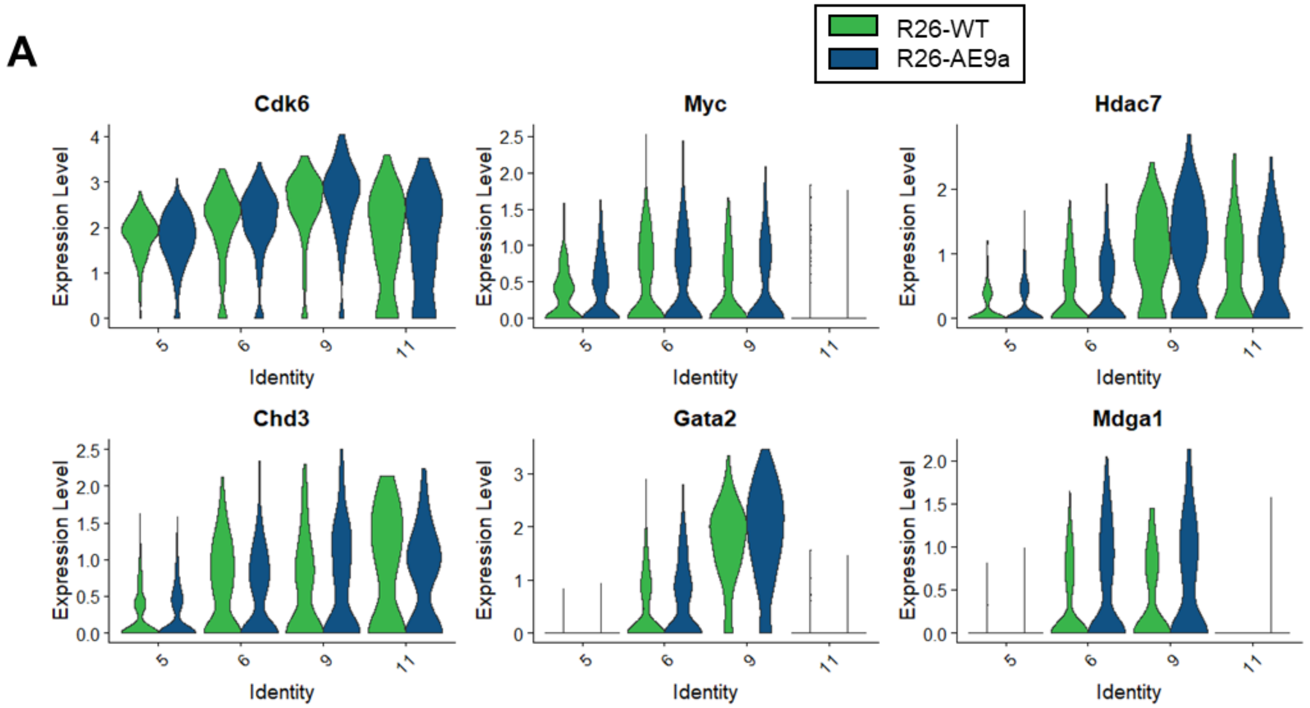

Supplement: Supplementary file 1 — Yan_et al_AE9a_Revised Supplementary Data_2023.pdf [file 41375_2023_2063_MOESM1_ESM.pdf]
